# Genetic interaction between *GL15* and *FDL1* modulates juvenile cuticle deposition and leaf permeability in maize

**DOI:** 10.1093/jxb/eraf265

**Published:** 2025-06-18

**Authors:** Giulia Castorina, Frédéric Domergue, Gabriella Consonni

**Affiliations:** Dipartimento di Scienze Agrarie e Ambientali (DiSAA), Università Degli Studi di Milano, Milan, Italy; CNRS, LBM, UMR 5200, Université de Bordeaux, Villenave d'Ornon F-33140, France; Dipartimento di Scienze Agrarie e Ambientali (DiSAA), Università Degli Studi di Milano, Milan, Italy; Max Planck Institute of Molecular Plant Physiology, Germany

**Keywords:** Cuticle, cutin, drought tolerance, epidermal layer, FUSED LEAVES1, GLOSSY15, juvenile traits, leaf permeability, waxes, *Zea mays*

## Abstract

The plant cuticle is a hydrophobic layer produced by the epidermis of primary aerial tissues that serves as the primary barrier between the plant surface and the external environment, whose main function is to limit water loss. This study investigated the roles and interactions between the regulatory genes *ZmFDL1* and *ZmGL15* in modulating juvenile cuticle deposition and function in maize. Expression and lipid analyses, morphological studies, and permeability assays were performed on single and double mutants. Our results showed an additive effect of ZmFDL1 and ZmGL15 transcription factors on wax abundance and an epistatic effect of *gl15-S* on *fdl1-1* in determining cutin deposition. *ZmFDL1* has a key role in controlling juvenile cuticle deposition and preventing water loss, while the main role of *ZmGL15* is to maintain a juvenile cuticle. Lack of ZmGL15 activity, as observed in the *gl15-S* mutant, results in the acquisition of a cuticle characterized by a higher cutin content, with increased ω-hydroxy fatty acids (FAs) as well as polyhydroxy FAs, and a lower wax content, with a decrease in both aldehydes and long-chain alcohols. These changes result in an increased water-holding capacity of the seedlings under drought stress conditions. Furthermore, *gl15-S* has an epistatic effect on the phenotype of the *fdl1-1* mutant. In the double *fdl1-1 gl15-S* mutant, the absence of ZmGL15 activity mitigates the *fdl1-1* morphological abnormalities and rescues the increased *fdl1-1* cuticle-mediated leaf permeability.

## Teaser

Detailed phenotypic and lipidomic analyses of mutants in *ZmFDL1* and *ZmGL15* reveal that the two transcription factors genetically interact in determining cuticle deposition in maize seedling, affecting both wax and cutin load and composition.

## Introduction

The plant cuticle is a hydrophobic layer produced and secreted by shoot epidermal cells which forms the main barrier between the plant aerial surfaces and the external environment. The cuticle limits non-stomatal water loss and protects plants against numerous abiotic and biotic stresses, including drought, UV radiation, heat, and pest and pathogen invasion (Yeats and Rose, 2013; Xue *et al*., 2017; Ziv *et al*., 2018; Batsale *et al*., 2021).

Cutin and waxes are the two principal lipid components of the cuticle. Cutin, a cross-esterified polymer of hydroxylated and epoxy-long-chain (16 and 18 carbon atoms in length) fatty acids (FAs; Li-Beisson *et al*., 2013; Fich *et al*., 2016), forms an insoluble matrix that is embedded by cuticular waxes. Cuticular waxes, mainly produced from very-long-chain fatty acyl-CoAs of ≥20 carbon atoms in chain length, are a complex mixture of acids, alcohols, aldehydes, alkanes, ketones, and wax esters, but can also contain variable amounts of cyclic compounds such as triterpenoids and phenylpropanoids (Bernard and Joubès, 2013; Lee and Suh, 2015). Cuticular waxes are also layered on top of the cutin matrix, forming epicuticular films or wax crystalloids (Bernard and Joubès, 2013; Yeats and Rose, 2013).

Cuticle composition and structure vary among plant species and organs of a single plant, as well as across developmental stages. In maize, cuticle composition and structure are among the traits that distinguish the leaves of juvenile and adult vegetative phases. In most genetic backgrounds, the first 4–5 leaves are juvenile leaves (Freeling, 1992). They have a thin cuticle and are densely covered with epicuticular wax crystals (Sylvester *et al*., 1990; Bongard-Pierce *et al*., 1996). Among them, the first two leaves, also called seedling leaves, express all the juvenile epidermal traits but are morphologically and anatomically distinct from the other juvenile leaves and have a different pattern of gene expression (Beydler *et al*., 2016; Poethig and Fouracre, 2024).

Waxes of juvenile leaves mainly contain very-long-chain alcohols and aldehydes, and a lower proportion of alkanes and esters (Bianchi *et al*., 1978; Sturaro *et al*., 2005; Javelle *et al*., 2010; Loneman *et al*., 2017). Then, approximately from leaf 5 to leaf 6, we found transition leaves which display a mosaic of juvenile and adult traits, the leaf tip being juvenile while the base has adult characteristics, in a pattern reflecting basipetal differentiation (Lawson and Poethig, 1995; Bongard-Pierce *et al*., 1996; Kerstetter and Poethig, 1998). The following leaves up to the vegetative-to-reproductive transition are adult leaves. Adult leaves have a thick cuticle, an amorphous wax layer on their surfaces, with alkanes and alkyl esters as principal wax components (Bianchi *et al*., 1984; Avato *et al*., 1990; Yang *et al*., 1993; Bourgault *et al*., 2020).

Aliphatic cutin composition in juvenile leaves is characterized by high concentrations of ω-hydroxy FAs, polyhydroxy FAs, and very-long-chain FAs (VLCFAs) (Castorina *et al*, 2020; Chen *et al*., 2024). In adult leaves, aliphatic cutin is mainly composed of dihydroxyhexadecanoic acid and typical members of the C18 family of cutin acids, including hydroxy and hydroxyepoxy acids (Bourgault *et al*., 2020; Chen *et al*., 2024). In addition, maize cutin was shown to contain at both stages very high amounts phenolic compounds such as ferulic, caffeic, and coumaric acids (Bourgault and Molina, 2025; Chen *et al*., 2024). Finally, an increase in the abundance of cutin monomers has been observed along the length of the leaf during acquisition of adult identity (Bourgault *et al*., 2020).

Because of its protective functions, the cuticle is considered as a promising target for improving plant tolerance to environmental stresses, particularly drought. Cuticle composition and structure have been shown to influence cuticular permeability and, in turn, drought tolerance in various plant species. Genetic variants with defective cuticle composition have been frequently associated with increased cuticular permeability and reduced drought tolerance (Park *et al*., 2010; Zhou *et al*., 2013; Zhu and Xiong, 2013; Li *et al*., 2019), whereas overexpression of regulatory or biosynthetic genes of the cuticle biosynthetic pathways have been shown to cause increases in drought tolerance (Aharoni *et al*., 2004; Zhang *et al*., 2007; Kosma *et al*., 2009; Cui *et al*., 2016). Moreover, the expression of regulatory genes, which stimulates the activation of cuticle biosynthesis-related genes, was shown to be promoted by drought (Seo *et al*., 2009, 2011; Lee and Suh, 2015; Bi *et al*., 2016).

In maize, the characterization of mutants in *ZmGLOSSY15* (*ZmGL15*), *ZmGLOSSY3* (*ZmGL3*), *ZmMYB94*/*FUSED LEAVES1* (*ZmFDL1*), and *ZmMYB84* suggested a regulatory role for the products of these gene (Moose and Sisco, 1994; Liu *et al*., 2012; La Rocca *et al*., 2015; Liu *et al*., 2022). In addition, overexpression of *ZmOUTER CELL LAYER1* (*ZmOCL1*) in maize plants revealed a link between this epidermis-specific homeodomain-leucine zipper IV transcription factor and cuticle deposition/biosynthesis (Javelle *et al*., 2010). In previous studies, we have characterized the ZmMYB94/FUSED LEAVES1 (ZmFDL1) transcription factor as a key regulator of cuticle deposition in maize seedlings (La Rocca *et al*., 2015; Castorina *et al*., 2020). Seedlings lacking ZmFDL1 show a decrease in epicuticular wax load, mainly due to a reduction in primary very-long-chain alcohols, as well as in the cutin content, which is mainly attributed to decreases in ω-hydroxy FAs and polyhydroxy FAs. These cuticle defects impact seedling development and leaf water-holding capacity (La Rocca *et al*., 2015; Castorina *et al*., 2020).

*ZmGLOSSY15* (*ZmGL15*), an *APETALA-2* like gene, was shown to play a fundamental role in promoting epidermal juvenile leaf identity traits and suppressing adult leaf identity traits (Moose and Sisco, 1994, 1996). *ZmGL15* is controlled by *miR172* that, by down-regulating *ZmGL15*, promotes the transition from juvenile to adult vegetative phase (Lauter *et al*., 2005). In *gl15* mutant plants, the dull appearance of wild-type juvenile leaves is replaced by the glossy phenotype, which is characteristic of adult leaves.

Previously, we reported that *ZmFDL1* and *ZmGL15* showed similar variations in their expression levels in response to drought, as both genes were up-regulated under conditions of water scarcity (Castorina *et al*., 2020). In the present study, we provide a comprehensive picture of the role of the *ZmGL15* gene in controlling chemical composition, structure, and function of the juvenile maize cuticle. We further show that these cuticle-related traits are controlled by a genetic interaction between *ZmFDL1* and *ZmGL15*. In maize seedlings, cuticle features of juvenile leaves, as well as the extent of cuticle-mediated leaf permeability, depend on the allelic constitution of both *ZmFDL1* and *ZmGL15* genes and on their combination. Moreover, data obtained from the chemical analysis of wild-type and single and double mutant genotypes allowed the identification of some correlations between variation in cuticle components and changes in cuticle-mediated leaf permeability. Overall, these results provide new insights into the identification of the cuticle components that provide better protection against water loss. This information may be useful to generate new strategies for improving plant response to drought stress as well as plant adaptation to water scarcity conditions.

## Materials and methods

### Plant materials and growth conditions

The maize *gl15* mutant (*gl15-Sprague*, *gl15-S*) seeds were obtained from the Maize Genetics COOP Stock Center (catalog no. 917E; http://maizecoop.cropsci.uiuc.edu) (Portwood *et al*., 2019 ). The other *gl15* mutant alleles [*gl15-Hayes* (*gl15-H*) and *gl15-Lambert* (*gl15-L*)] were kindly provided by Stephen Moose and were previously detailed (Moose and Sisco, 1994, 1996; Lauter *et al*., 2005). The *gl15* mutants were backcrossed three times to the B73 inbred line. In all the experiments performed, homozygous mutants and their wild-type control plants were from families segregating for a *gl15* allele.

The previously described maize (*Zea mays*) *fdl1-1* mutant in the B73 inbred line (Castorina *et al*., 2020), used in this work, was originally identified in the selfed progeny of a maize line crossed as female to an *En/Spm* line (La Rocca *et al*., 2015). To generate double-homozygous *fdl1-1 gl15-S* plants, single-homozygous *fdl1-1* and *gl15-S* plants were crossed. Double-heterozygous F_1_ plants were then selfed to generate segregating families in which single- and double-homozygous mutants were identified. In all the experiments performed, homozygous mutants and their wild-type control plants were from the same segregating family. Plants were grown in a growth chamber with controlled temperature (25 °C night/30 °C day) under a long-day photoperiod (16 h light/8 h dark) and with a photon fluence of 270 µmol m^−2^ s^−1^.

To evaluate *gl15-S* plant response to water stress, a drought stress experiment was conducted. Maize seedlings were germinated in truncated cone-shaped net pots (72 ml) and initially grown under well-watered (WW) conditions. After plant establishment, the drought was induced by completely withholding irrigation in seedlings 17 days after sowing (DAS) (time point 0) and no additional water was supplied for the entire duration of the experiment. The small volume of the pots promotes uniform and rapid soil drying. The endpoint of the experiment was defined by visible signs of wilting. This method allows for the quantification of drought-induced changes in plant water status and biomass allocation, as described in previous studies (Verslues *et al*., 2006; Skirycz and Inzé, 2010). Leaf relative water content (RWC) was measured 24 h and 48 h later. Then, the drought was prolonged for an additional 5 d up to the appearance of the severe wilted phenotypes. In parallel, control plants were grown in WW conditions during the whole experiment. To measure the impact of the stress on plant growth, the epigeal organs of the seedlings were dried at 60 °C for 4 d and the DW was measured.

### Epidermal cell density and stomatal index

Epidermal cell density and stomatal index were measured using the surface imprint method on the abaxial and adaxial sides of the mid portion of fully expanded leaves, as previously described (Castorina *et al*., 2018). We analyzed the mid portion of the second and third fully expanded leaves of wild-type and *gl15-S* mutant plants. The stomatal index was determined as [number of stomata/(number of epidermal cells+number of stomata)]×100.

### Toluidine blue staining of maize epidermal peels for phase transition analysis

Epidermal peels were sampled by hand from the mid portion of the second, third, and fourth leaves of wild-type, *gl15-S*, *fdl1-1*, and *fdl1-1 gl15-S* plants. The staining was performed as in the protocol described by Dudley and Poethig (1993) with slight modifications as follows: fresh tissues were soaked in tap water and then stained for 5 min in a 1:1 (v/v) aqueous solution of 0.05% (w/v) toluidine blue O and 0.1 M phosphate buffer at pH 6. The stained peels were washed for 1 min in tap water and photographed using a light microscope (Olympus BX50) at ×40 magnification. Juvenile and adult epidermal cells stained violet/pink or clear/aquamarine, respectively.

### Cuticular cutin and wax analysis

Cuticle analyses were performed in the first, second, and third mature leaves of 20-day-old homozygous *gl15-S* and its wild-type control seedlings, as well as in the second fully expanded leaves of 15-day-old double-homozygous *fdl1-1 gl15-S*, single-homozygous *fdl1-1* and *gl15-S*, and wild-type plants. Six independent biological replicates per leaf, each of which consisted of a pool of two leaves, were analyzed per genotype. Fresh leaves were harvested, weighed (FW), and immediately used, first for extraction of epicuticular waxes, and then for cutin analysis.

For wax analysis, epicuticular waxes were extracted by immersing leaves for 30 s in chloroform containing docosane as an internal standard. Extracts were dried under N_2_ gas and derivatized by heating at 110 °C for 15 min in 150 µl of *N*,*O*-bis(trimethylsilyl)trifluoroacetamide):trimethylchlorosilane (BSTFA-TMCS; Sigma). After evaporation of the surplus of derivatizing agent, silylated samples were dissolved in hexane and analyzed by GC with an Agilent 7890B gas chromatograph equipped with a flame ionization detector and a HP-5MS column (30 m×0.25 mm×0.25 µm) with helium as the carrier gas. The initial temperature of 50 °C was held for 1 min, increased at 25 °C min^−1^ to 150 °C, held for 2 min at 150 °C, increased again at 10 °C min^−1^ to 320 °C, and held for 6 min at 320 °C. Injector and detector temperatures were set at 250 °C. Quantification was based on flame ionization detector peak areas and the internal standard, and expressed in µg g^–1^ FW. Molecular identities were determined using an Agilent 6850 gas chromatograph equipped with the same column and an Agilent 5975 mass spectrometric detector (70 eV; mass-to-charge ratio of 50–750). The same GC program was used, with helium (1.5 ml min^−1^) as carrier gas. Also, the presence of wax esters was checked using a ‘long’ GC program (with a final 25 min step at 320 °C), but they could not be detected in any sample, indicating that these minor components were below the detection limit of our procedure.

For cutin analysis, dewaxed leaves were immediately immersed in hot isopropanol for 30 min at 85 °C and, after cooling, the solvent was discarded. The residue was further extensively delipidated by extracting the soluble lipids for 24 h with successively 2.5 ml of CHCl_3_:CH_3_OH (2:1, v/v), 2.5 ml of CHCl_3_:CH_3_OH (1:1, v/v), 2.5 ml of CHCl_3_:CH_3_OH (1:2, v/v), and 2.5 ml of CH_3_OH, all performed at room temperature on a wheel rotating at 33 rpm. Residues were dried in a fume hood at room temperature for 2 d and then in a desiccator for another 2 d. Dried residues were weighed, and 10–30 mg of each sample was depolymerized by transmethylation at 85 °C for 3 h in 1 ml of 5% (v/v) sulfuric acid in methanol containing 5 mg each of heptadecanoic acid (C17:0), ω-pentalactone (C15ωOH), and pentadecanol (C15:0-OH) as internal standards. After cooling, 1 ml of NaCl (2.5%, w/v) was added, and the released monomers were extracted with chloroform. Extracts were washed once with 1 ml of saline solution [NaCl 0.09% (v/v) in 100 mM Tris, pH 8.0] and dried under a gentle stream of nitrogen. Samples were silylated and analyzed using GC-MS as described for wax analysis. Quantification of FAs, hydroxyl acids, and fatty alcohols was based on peak areas and the respective internal standards (C17:0, C15ωOH, or C15:0-OH).

### Chlorophyll leaching assay and toluidine blue assay

The chlorophyll leaching assay was performed on mutant and wild-type first, second, third, and fourth fully expanded leaves from several independent biological replicates per genotype. Leaf samples were dissected into pieces of 8 cm in length measured from the apex, weighed, immersed in 80% (v/v), ethanol and incubated in the dark at room temperature. Chlorophyll released in the solution was quantified by measuring the absorbance at 647 nm and 664 nm with a spectrophotometer (Cary 60 UV-Vis, Agilent Technologies) until chlorophyll extraction was complete. The concentration of chlorophyll was calculated using the equation described by Lolle *et al*. (1997): total micromoles of chlorophyll=7.93*×A*_664_+19.53*×A*_647_. Also, the data obtained were normalized per gram of FW and expressed as a percentage of total chlorophyll. Biological replicates consisted of leaves taken from independent plants.

For the toluidine blue permeability test, 3-day-old etiolated coleoptiles or mature leaves were stained in the dark for 5 min and 24 h, respectively, in a toluidine blue solution (0.05% w/v) with Tween-20 (0.1% v/v), and washed in tap water, as previously described (Tanaka *et al*., 2004; Doll *et al*., 2020). For quantification, coleoptiles were excised, placed in tubes containing 1 ml of 80% ethanol, and incubated for 4 h in the dark until all dye and chlorophyll had been extracted. Absorbance of the solution was detected at 626 nm and 430 nm using a spectrophotometer. Several biological replicates, each consisting of a single coleoptile, were assessed per genotype.

### Water loss and leaf relative water content

To determine the rate of the leaf and seedling water loss, plants were first adapted for 2 h in the dark before the entire seedlings or the leaves were detached and weighed to determine the initial FW. Sample weights were then estimated at designated time intervals, and water loss was calculated as the percentage of the initial FW. The experiment was conducted on the laboratory bench at room temperature (18–25 °C) and several biological replicates were measured for each genotype.

To monitor the leaf moisture content under drought stress, the RWC of the leaf was measured in wild-type control and *gl15-S* mutant plants according to Smart and Bingham (1974). The second and third leaf RWC was measured at 0, 24, and 48 h after withholding irrigation, taking a leaf blade disc of 0.5 cm^2^ from the median portion of the leaf. FW was determined immediately after sample collection. The total weight (TW) was obtained by soaking the tissue for 24 h in distilled water. Then, the discs were fully dried and, eventually, the DW was measured. The leaf RWC was calculated with the following formula: RWC=([FW−DW]/[TW−DW])×100. For each treatment, the leaf RWC was measured in a minimum of four independent plants per genotype.

### SEM analyses of epicuticular waxes

For the analysis of epicuticular waxes by SEM, samples were air-dried and processed according to Castorina *et al.* (2018, 2023). Using double-sided tape, leaf pieces from the mid portion were fixed to a carbon base on aluminum stubs (ø 12.5 mm; 3.2×8 mm pin) and sputter coated with gold using a Scancoat Six Sputter Coater (Edwards). Micrographs of the abaxial and adaxial leaf surfaces were acquired with an SEM-EDS JSM-IT500 LV electron microscope (JEOL Spa).

### RNA extraction and gene expression analysis

Total RNA was extracted with the TRIzol Reagent (Life Technologies), suspended in RNase-free milliQ dH_2_O, and treated with RQ1 RNase-Free DNase (Promega). RNA concentration was measured with the NanoDrop^®^ ND-1000 spectrophotometer (Thermo Scientific). First-strand cDNA was synthetized using the High-Capacity cDNA Reverse Transcription Kit (Thermo Fisher Scientific) from 1000 ng of total RNA, according to the manufacturer's instructions. Quantitative real-time PCR (RT-qPCR) was performed with the 7300 Real-Time PCR System (Applied Biosystems), using GoTaq qPCR Master Mix (Promega). A no-template control was also included in each run for each gene. Each sample was conducted in two technical replicates and three biological replicates. Relative gene expression was calculated by applying the 2^−ΔΔCt^ method (Livak and Schmittgen, 2001) by averaging the values of biological replicates. Data were normalized to the reference *ZmEF1α* (Zm00001eb385900) gene, which is known as a reliable reference gene for overall gene expression normalization in maize and has stable expression under comparable experimental conditions (Lin *et al*., 2014). The gene-specific primers are listed in Supplementary Table S1.

To evaluate tissue-specific gene expression, a tissue-specific enrichment assay was performed. B73 wild-type seedlings were grown on soil and the second leaf was sampled at 10 DAS. The epidermis was manually peeled off with the help of tweezers and immediately frozen in liquid nitrogen. From the remaining portion of the leaf, the green tissues, consisting mostly of mesophyll, were sampled with the help of a spatula. Also, the whole leaf was sampled. Four biological replicates were sampled per tissue and each replicate consisted of leaf tissues from three independent plants. These enriched-tissue samples were then subjected to RNA extraction and RT-qPCR as described above.

### Statistical analysis

All data were statistically analyzed. Student’s *t*-test (ns, not significant, *P<0.05, **P<0.01, ***P<0.001, and ***P<0.0001) or ANOVA (*P*<0.05), combined with post-hoc Tukey tests, was conducted using the statistical packages Prism GraphPad 10.

### Accession numbers

The gene sequences from this article can be found in the Maize Genetics and Genomics Database (MaizeGDB) or GenBank/EMBL databases under the following accession numbers: Zm00001eb328280 (*ZmFDL1/MYB94*), Zm00001eb387280 (*ZmGL15*), Zm00001eb042160 (*ZmHTH1*), Zm00001eb311010 (*ZmONI3*), Zm00001eb195850 (*ZmGL3*), Zm00001eb246270 (*ZmGL8*), Zm00001eb190120 (*ZmGL4*), Zm00001eb018600 (*ZmKCS39*), Zm00001eb296230 (*ZmKCS16*), Zm00001eb176110 (*ZmCER4*), Zm00001eb247450 (*ZmCER1*), Zm00001eb156680 (*ZmWSD1*), Zm00001eb313510 (*ZmGL1*), Zm00001eb071110 (*ZmGL2*), Zm00001eb122470 (*ZmGL13*), Zm00001eb087050 (*ZmGL14*), and Zm00001eb385900 (*ZmEF1α*).

## Results

### Spatiotemporal expression analysis of *ZmFDL1* and *ZmGL15* genes

**Fig. 1.**
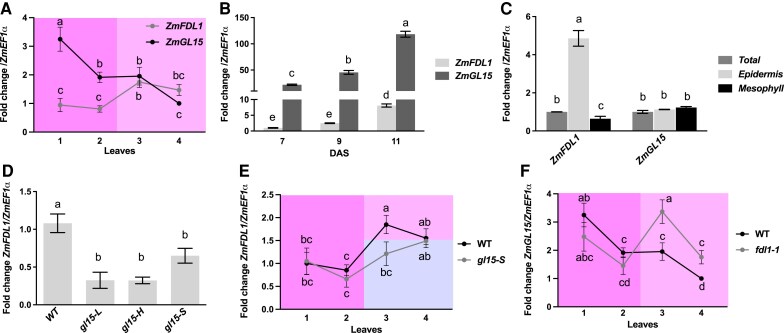
Gene expression analysis of *ZmGL15* and *ZmFDL1*. Transcript level quantification performed by RT-qPCR. *ZmGL15* and *ZmFDL1* gene expression analysis (A) in the first, second, third, and fourth leaves of wild-type plants sampled at 5, 7, 10, and 14 DAS, respectively, (B) in the second leaf of wild-type plants at 7, 9, and 11 DAS, and (C) in the whole leaf (Total), mesophyll, and epidermal tissues from the second leaves of 10 DAS wild-type plants. (D) Gene expression level of *ZmFDL1* in homozygous *gl15* mutants and wild-type (WT) control seedlings at the coleoptile developmental stage. Transcript level quantification of (E) *ZmFDL1* and (F) *ZmGL15* genes in WT control and homozygous *gl15-S* or *fdl1-1* mutant leaves, respectively. Values represent the mean fold change variations of a minimum of four biological replicates. Error bars are ±SE. Different letters denote significant differences assessed by Tukey’s HSD test (*P*<0.05). In (A), (E), and (F), the graph background colors mark the leaf phase identity. Pink and light blue shades highlight the juvenile and adult vegetative phase, respectively. Darker pink marks juvenile seedling leaves.

We have previously reported that *ZmFDL1* and *ZmGL15* transcription factor genes are both expressed in maize seedling and are drought responsive (Castorina *et al*., 2020). To further define their pattern of expression, RT-qPCR was performed (Fig. 1).

The transcript levels of *ZmFDL1* and *ZmGL15* were investigated in the first, second, third, and fourth leaves collected from wild-type seedlings grown for 5, 7, 10, and 14 d, respectively. While the expression of *ZmFDL1* slightly increased, that of *ZmGL15* steadily decreased from the first to the fourth leaf (Fig. 1A). Instead, throughout the development of the second leaf, which was collected at 7, 9, and 11 DAS from wild-type seedlings, the transcript levels of both *ZmFDL1* and *ZmGL15* showed a progressive increase, and the expression level of *ZmGL15* was much higher than that of *ZmFDL1* (Fig. 1B).

We performed an expression enrichment analysis in which different tissues from the mature second leaf of 10 DAS seedlings were sampled. We observed that the *ZmFDL1* transcript level was significantly higher in the sample enriched in epidermis as compared with the mesophyll-enriched and whole-leaf samples (Fig. 1C). Despite epidermis and mesophyll samples probably being contaminated by other tissue layers, this approach indicated that *ZmFDL1* is likely to be preferentially expressed in the epidermal layer. This observation is consistent with the pattern observed by *in situ* hybridization assays (Liu *et al*., 2021 ) and is consistent with *ZmFDL1*-specific involvement in the control of cuticle deposition, a process which occurs exclusively in epidermal cells. Moreover, this result confirmed the robustness of our expression enrichment approach. No differences were detected among whole-leaf, mesophyll, and epidermal tissue samples for the transcript levels of *ZmGL15* (Fig. 1C), suggesting that this gene does not exhibit preferential expression in a specific tissue layer of the leaf.

The ZmFDL1 transcription factor is a key regulator of cuticle deposition in juvenile vegetative tissues. Lack of ZmFDL1 activity affects the expression of several cuticular wax genes involved in different modules of the biosynthetic pathway (Castorina *et al*., 2020). Among the cuticle-related genes which were shown to be differentially expressed in *fdl1-1* mutant seedlings (Castorina *et al*., 2020), 14 were analyzed for their expression in epidermis- and mesophyll-enriched samples (Supplementary Fig. S1). Suh *et al.* (2005) employed a comparable approach, which involved the analysis of RNAs extracted from manually peeled stem epidermis in Arabidopsis. Their aim was to identify genes involved in the biosynthesis of waxes and cutin. It is important to note that in our experiment we were able to show that the expression level of most of these genes was significantly higher in the samples enriched in epidermal tissue (Supplementary Fig. S1).

We previously showed that the expression of *ZmGL15* was lower in the *fdl1-1* mutant compared with the wild type in seedlings at the coleoptile developmental stage (Castorina *et al*., 2020). In this work, a significant down-regulation of *ZmFDL1* was observed in coleoptiles of three homozygous *gl15* genotypes that carried different *ZmGL15* mutant alleles, namely *gl15-L*, *gl15-H*, and *gl15-S* (Fig. 1D). This indicated that the presence of a functional *ZmGL15* is necessary to promote the expression of *ZmFDL1* at the coleoptile stage of seedling development (Fig. 1D). At later developmental stages, the transcript level of *ZmFDL1* was similar in the first two juvenile seedling leaves of both *gl15-S* and the wild-type control but reduced in the third leaf, which is the first glossy and adult-like leaf of the *gl15-S* mutant compared with the wild type (Fig. 1E). Similarly, *ZmGL15* expression in the first two juvenile leaves of *fdl1-1* seedlings did not statistically differ from that in the wild type, even if it appeared to be slightly down-regulated (Fig. 1F). In the third and fourth leaves, *ZmGL15* transcript levels were higher in *fdl1-1* compared with wild-type plants (Fig. 1F). Collectively, these results suggest a complex interplay and fine-tuned regulation between vegetative phase identity and cuticle deposition.

### Analysis of cuticle-related leaf traits in *gl15-S* mutant plants

**Fig. 2.**
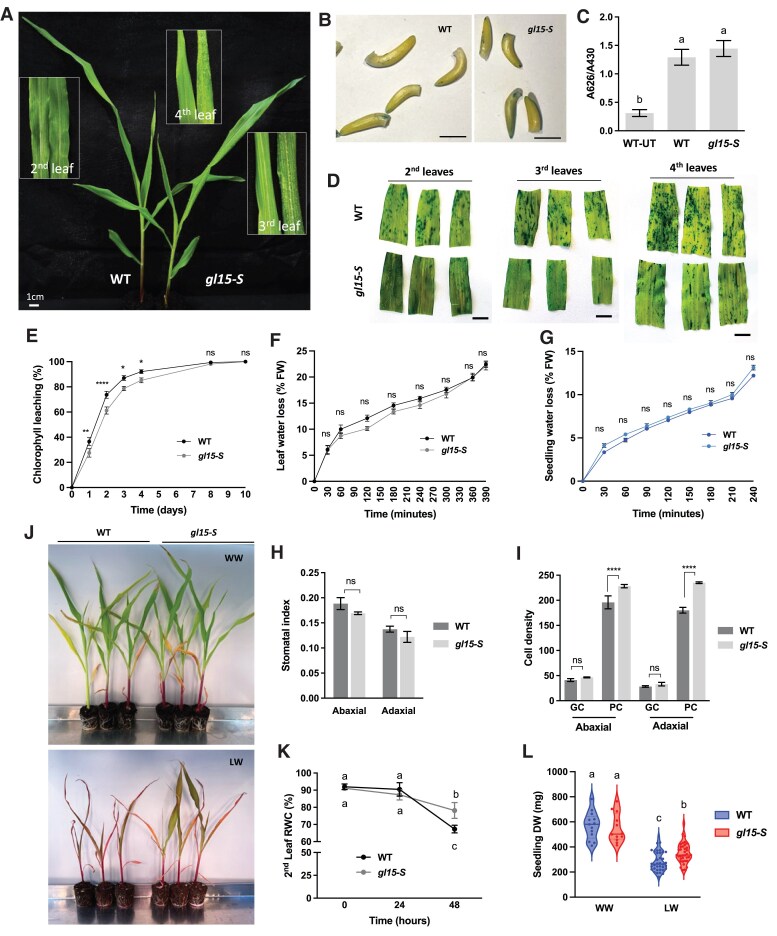
Cuticle-dependent phenotype in *gl15-S* and wild-type plants. (A) Representative image of wild-type (WT) and homozygous *gl15-S* 15-day-old seedlings. The insets represent a magnification of WT (left) and *gl15-S* (right) second, third, and fourth leaves. The glossy phenotype and the ability of the leaf surface to retain water drops were visible starting from the third leaf of *gl15-S* mutant plants. (B) Representative of toluidine blue-stained etiolated coleoptiles of 3 DAS WT and *gl15-S* plants (Bars, 1 cm) and (C) quantification of toluidine blue uptake, normalized to chlorophyll content. Values represent the average of minimum 10 biological replicates. Error bars represent ±SE. UT, untreated control. Different letters denote significant differences assessed by ANOVA (*P*<0.05). (D) Toluidine blue assay in the second, third, and fourth fully expanded leaves of WT and *gl15-S* mutant seedlings. Bars, 1 cm. (E) Cuticle-dependent leaf permeability assessed with the chlorophyll leaching assay in the second fully expanded leaf of 15-day-old WT and homozygous *gl15-S* plants. Values represent the mean of 10 biological replicates per genotype. Error bars are ±SE. (F) Leaf and (G) seedling transpiration, expressed as the percentage of water loss of the initial FW, was determined on the second fully expanded leaf and the epigeal organ of 15-day-old WT and homozygous *gl15-S* plants, respectively. Values represent the mean of a minimum of eight biological replicates per genotype. Error bars are ±SE. Differences were assessed by the Student’s *t*-test (*P<0.05; **P<0.01; ****P<0.0001; ns, not significant). (H) Stomatal index and (I) the density per 1 mm^2^ of guard cells (GCs) and pavement cells (PCs) were measured in both the abaxial and adaxial sides of the second leaf of WT and *gl15-S* plants. Values are the mean ±SE, and differences were evaluated by Student’s *t*-test. (J) Representative image of WT and homozygous *gl15-S* 21-day-old seedlings grown in well-watered (WW) conditions or subjected to drought stress (low-watered, LW). (K) The relative water content (RWC) was measured in the second leaf of WT and *gl15-S* plants subjected to 24 h and 48 h of water scarcity imposed by withholding irrigation. Values represent the mean of a minimum of four biological replicates. Error bars are ±SE. Different letters denote significant differences assessed by Tukey’s HSD test (*P*<0.05). (L) Plant biomass of WT and homozygous *gl15-S* seedlings grown in WW (*n*=12) and LW (*n*=36) conditions, respectively. Values represent the mean of biological replicates (n). Error bars are ±SE. Different letters denote significant differences assessed by Tukey’s HSD test (*P*<0.05).

Mutations in the *ZmGL15* gene were shown to cause the precocious appearance of adult epidermal features (Moose and Sisco, 1994). Accordingly, we observed the precocious presence of a glossy phenotype in *gl15-S* mutant seedlings, which was evident starting from the third leaf (Fig. 2A). Third and fourth leaves of *gl15-S* seedlings appeared glossy, or shiny green, in contrast to the dull appearance of the corresponding wild-type leaves (Fig. 2A). In addition, when leaves were sprayed with water, water droplets adhered to *gl15-S* mutant leaf surfaces whereas the water slipped away on wild-type leaves (Fig. 2A, insets). More precisely, the glossy phenotype showed a distal to proximal gradient in the third leaf of the *gl15-S* mutant. The proximal-to-middle portion of the third leaf displayed an obvious glossy phenotype, while the middle-to-distal leaf portion showed a decrease in the glossy phenotype intensity and the leaf tip appeared dull. This suggests that the third leaf is undergoing the transition from juvenile to adult phase. In addition, we analyzed the epidermal traits of the adaxial and abaxial sides of second and third leaves in the middle portion of the wild-type and *gl15-S* mutant plants. Juvenile epidermal traits (i.e. cells with wavy walls as well as a pink coloration of epidermal peels following toluidine blue staining) were observed in both leaves of wild-type plants (Supplementary Fig. S2A–D, I–L) and in the second leaf of *gl15-S* mutant plants (Supplementary Fig. S2E–H). In contrast, *gl15-S* mutant plants expressed both juvenile and adult epidermal cell traits, the latter including longer epidermal cells with highly crenulated lateral walls (Supplementary Fig. S2M, O) and aqua-/turquoise-colored areas in the toluidine blue-stained epidermal peels (Supplementary Fig. S2N, O). The results confirmed that the second leaf of the *gl15-S* mutant was juvenile while the third leaf was in transition, showing epidermal cells with traits of both phases.

We also quantified the accumulation of toluidine blue stain in coleoptiles, and no differences were detected in the *gl15-S* mutant relative to the wild type, suggesting no alteration in cuticle deposition during embryogenesis (Fig. 2B, C). However, the analysis of the second, third, and fourth fully expanded leaves showed differences in the accumulation of the toluidine blue stain between *gl15-S* and wild-type samples. A reduced uptake of toluidine blue appeared very clear in the third and fourth leaves of the *gl15-S* mutant compared with the wild type, suggesting a decrease in cuticle-dependent leaf permeability (Fig. 2D). To further investigate the impact of the *gl15-S* mutation on the leaf cuticle, leaf permeability was assessed using the chlorophyll leaching assay (Fig. 2E; Supplementary Fig. S3). The first (Supplementary Fig. S3A), second (Fig. 2E), and third (Supplementary Fig. S3C) leaves of *gl15-S* seedlings released chlorophyll more slowly as compared with wild-type control siblings, indicating a lower cuticle-dependent leaf permeability, whereas no statistical differences were observed for the fourth leaf (Supplementary Fig. S3E). Then, a water loss time course experiment was performed on wild-type and *gl15-S* leaves by estimating the rate of leaf and seedling weight loss in relation to the initial FW. The resulting *gl15-S* mutant profiles were comparable with that of wild-type plants (Fig. 2F, G; Supplementary Fig. S3B, D, F), suggesting that the cuticle-dependent leaf permeability changes in *gl15-S* were not sufficient to also influence the leaf transpiration.

We then investigated the *gl15-S* leaf stomatal traits that could affect leaf transpiration. The stomatal index (Fig. 2H; Supplementary Fig. S3G) and stomatal density (guard cells; Fig. 2I; Supplementary Fig. S3H) were measured on both the abaxial and adaxial leaf side, and no statistical differences were detected. However, an increased number of pavement cells was found in the *gl15-S* mutant compared with wild-type plants (Fig. 2I; Supplementary Fig. S3H). Since the cuticle acts as the first line of defense against external factors, we evaluated if *gl15-S* cuticle could affect drought tolerance (Fig. 2J–M). We compared wild-type and *gl15-S* seedlings grown under WW and low-watered (LW) conditions. Leaf RWC analysis revealed differences between wild-type and *gl15-S* mutant plants (Fig. 2L; Supplementary Fig. S3I). After 48 h of water scarcity, the leaf RWC measured in the second (Fig. 2L) and third (Supplementary Fig. S3I) leaves was higher in the *gl15-S* mutant as compared with the wild type. Furthermore, after prolonged withholding of irrigation, wild-type seedlings appeared more wilted than *gl15-S* mutants (Fig. 2J). The measurement of the plant biomass confirmed that *gl15-S* mutant seedlings were able to grow more under drought stress compared with wild-type plants (Fig. 2M), suggesting an increased water-holding capacity that may be due to the reduced cuticle-dependent leaf permeability of *gl15-S* (Fig. 2D, E).

**Fig. 3.**
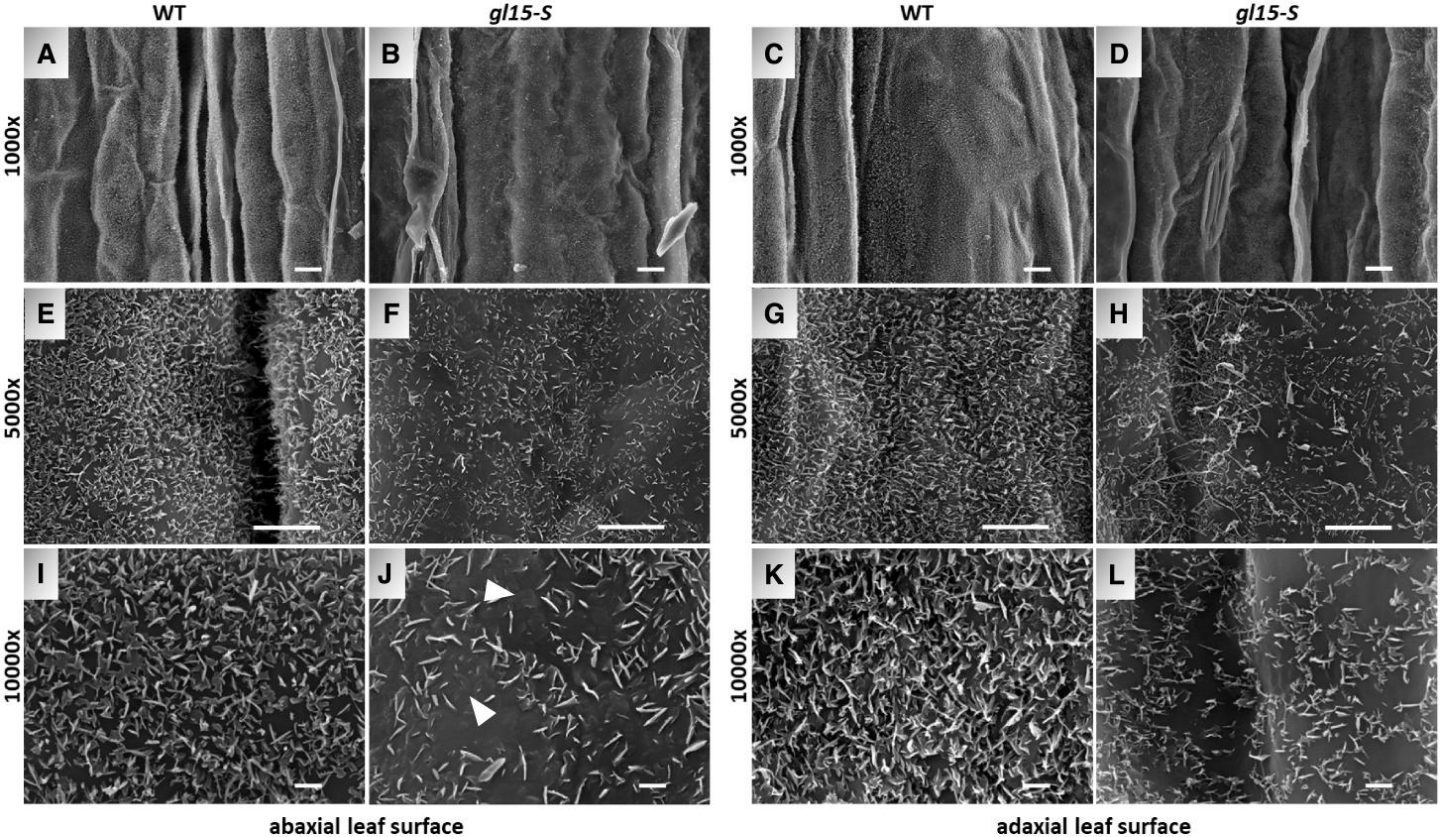
Distribution of cuticular waxes on the leaf surfaces of the *gl15-*S mutant. SEM micrograph images of both the abaxial and adaxial surface of the third fully expanded leaf in a wild-type (WT) control plant (A, E, I, C, G, K) and single homozygous *gl15-S* (B, F, J, D, H, L) mutant have been acquired at ×1000, ×5000, and ×10 000 magnification. Scale bars correspond to 10 µm (A–D), 5 µm (E–H), and 1 µm (I–L). White arrowheads highlight flattened and embedded wax crystalloids within the amorphous layer.

Finally, we examined whether the abundance and shape of epicuticular wax crystals were affected in the *gl15-S* mutant using SEM. These analyses were conducted using the mid portion of the third leaf of wild-type and *gl15-S* mutant plants (Fig. 3). Fewer wax crystals were observed on both abaxial (Fig. 3F, J) and adaxial (Fig. 3H, L) surfaces of the *gl15-S* mutant glossy leaves relative to the surfaces of the wild-type dull leaves (Fig. 3E, I, G, K). In addition, these crystalloids appeared much smaller, flattened, and embedded within the amorphous epicuticular film layer (Fig. 3F, J, H, L). A similar reduction in size and distribution of the epicuticular wax crystals was previously observed on glossy leaves of cuticle-related gene maize mutants (Beattie and Marcell, 2002; Li *et al*., 2019; Zheng *et al*., 2019; Liu *et al*., 2024).

Overall, these results confirmed that the *gl15-S* mutation causes an earlier adult transition and alterations in both structure and functions of the juvenile cuticle. Interestingly, *gl15-S* leaf permeability was lower and, under drought stress, *gl15-S* leaf RWC was higher compared with the wild type, thus suggesting that the *gl15-S* cuticle composition could be more efficient in preventing water loss.

### ZmGL15 regulates both cutin and wax deposition

**Fig. 4.**
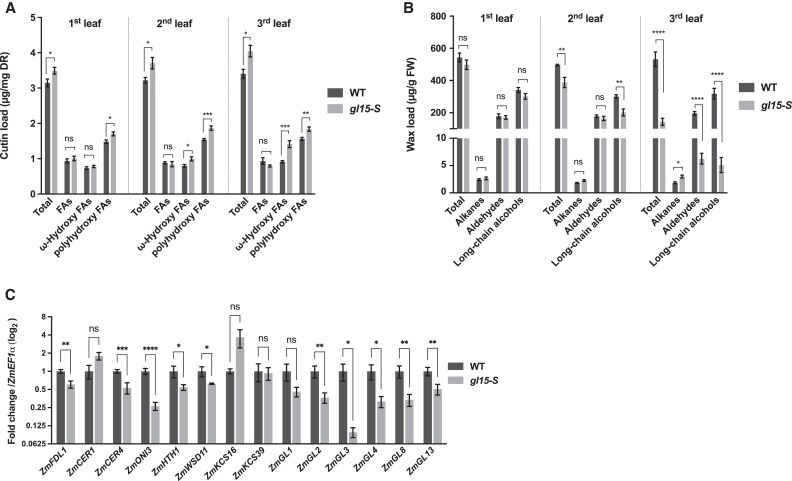
Cuticle composition in *gl15-S* plants. (A) Cutin aliphatic and (B) cuticular wax content of the first, second, and third fully expanded leaf from 21-day-old *gl15-S* and wild-type (WT) plants. Values represent the mean of six biological replicates per genotype ±SE. (C) Gene expression analysis of cuticle-related genes. The transcript levels of *ZmFDL1*, *ZmCER1*, *ZmCER4*, *ZmONI3*, *ZmHTH1*, *ZmWSD11*, *ZmKCS16*, *ZmKCS39*, *ZmGL1*, *ZmGL2*, *ZmGL3*, *ZmGL4*, *ZmGL8*, and *ZmGL13* genes were analyzed by RT-qPCR in the third leaf of homozygous *gl15-S* mutants and WT control plants. Values represent the mean fold change of four independent biological replicates. Error bars are ±SE. Comparison was made between genotypes, and significant differences were assessed by Student’s *t*-test (*P<0.05; **P<0.01; ***P<0.001; ****P<0.0001; ns, not significant).

To examine the effects of the *gl15-S* mutation on the cuticle composition of juvenile leaves, cutin and waxes from the first, second, and third leaves of *gl15-S* mutant and wild-type plants were analyzed by GC (Fig. 4) (Domergue *et al*., 2010; Bourdenx *et al*., 2011).

Considering total cutin monomers amounts, a higher cutin load was observed in all three examined leaves of the *gl15-S* mutants as compared with wild-type leaves (Fig. 4A). While no differences were detected in the content of FAs (Fig. 4A; Supplementary Fig. S4C), an increase in ω-hydroxy FAs as well as in polyhydroxy FAs (Fig. 4A) was observed in the *gl15-S* mutant compared with wild-type samples. The increase in ω-hydroxy FAs was mainly associated with a progressive increase in the accumulation of the 18:1 ω-hydroxy FA isomer (18:1ωOH; Supplementary Fig. S4D), while an increase in the content of several polyhydroxy FAs was observed in the three mutant leaves (Supplementary Fig. S4E). Also, considering relative abundance in total cutin content, a progressive decrease in the accumulation in FAs and an increase in ω-hydroxy FAs were observed in *gl15-S* mutant leaves (Supplementary Fig. S4A). For example, in the third leaf, the cutin of the *gl15-S* mutant contained 19.7% of FAs and 34.7% of ω-hydroxy FAs compared with 27.1% and 26.9% in the wild type, respectively (Supplementary Fig. S4A). The cutin analysis also revealed the presence of considerable amounts of phenolic compounds, such as coumarate, ferulate, and caffeate (Supplementary Fig. S4F), as already reported in juvenile leaves by Bourgault and Molina (2025). An increase only for the ferulate levels was detected in the *gl15-S* mutant in the third leaf compared with wild-type plants (Supplementary Fig. S4F).

Concerning cuticular waxes, no differences were detected in the first leaf (Fig. 4B), but the *gl15-S* mutation caused a slight and strong reduction in the total wax load in the second and third leaves, respectively. Among wax metabolite classes, decreases in alcohols in the second leaf, and in both aldehydes and alcohols in the third leaf (Fig. 4B), were observed. The analyses of individual wax metabolites revealed a statistically significant reduction in the C32 *n*-aldehyde content, which was observed only in the third leaf (ALD32; Supplementary Fig. S4H), and in the content of C32 primary alcohols (C32OH; Supplementary Fig. S4H), which was detected in both the second and third leaves. This change was greater in the third leaf, in which a reduction in C30 primary alcohols (C30OH; Supplementary Fig. S4I) was also observed. A slight increase in alkane content was in contrast observed in the third leaves (Fig. 4B), which was due to the higher accumulation of C31 and C33 alkanes (ALK31 and ALK33; Supplementary Fig. S4G). Considering relative abundance in total waxes, alkane and aldehyde increased, while long-chain alcohols decreased from the first to the third leaf of *gl15-S* mutant compared with wild-type seedlings (Supplementary Fig. S4B). In the third leaves, alkanes accounted for 23.8% and 3.5% of the total wax load, aldehydes for 43.3% and 37.3%, and alcohols for 32.9% and 59.2% in the *gl15-S* mutant and wild-type controls, respectively (Supplementary Fig. S4B).

These variations in cuticle composition indicated the involvement of ZmGL15 in the control of cuticle-related gene expression. This was confirmed by analyzing the expression levels of 14 cuticle-related genes, including *ZmFDL1*, in the third leaf of both the wild type and *gl15-S* mutants (Fig. 4C). Twelve out of 14 genes showed a significantly lower level of expression, while only two, *ZmCER1* and *ZmKCS16*, were up-regulated in the *gl15-S* mutant. Only the expression levels of *ZmKCS39* did not significantly differ. The transcript levels of the MYB regulatory genes *ZmFDL1* (La Rocca *et al*., 2015) and *ZmGL3* (Liu *et al*., 2012), and of the *ZmGL13* gene encoding an ABC transporter (Li *et al*., 2013), were down-regulated (Fig. 4C). Similarly, the expression of *ZmGL1* (Sturaro *et al*., 2005), *ZmGL2* (Tacke *et al*., 1995; Alexander *et al*., 2020), *ZmGL4* (Liu *et al*., 2009), and *ZmGL8* (Dietrich *et al*., 2005) genes, which are all involved in FA metabolism, seemed to be stimulated by ZmGL15, since they were down-regulated in *gl15-S* mutant leaves (Fig. 4C). The cutin-related *ZmONI3* and *ZmHTH1* genes, which are both presumably involved in the synthesis of α,ω-dicarboxylic acids, were also down-regulated (Fig. 4C). As to genes related to wax biosynthesis, *ZmCER4* and *ZmWSD11*, putatively involved in the synthesis of long-chain primary alcohols and of long-chain wax ester, respectively, were both down-regulated. In agreement with our wax analyses, the *ZmCER1* gene, which is involved in the production of long-chain alkanes, was up-regulated (Fig. 4C).

### Analysis of cuticle-dependent developmental defects shows that *gl15-S* is epistatic to *fdl1-1*

To examine the genetic relationship between *ZmGL15* and *ZmFDL1*, F_2_ progenies were produced from selfing heterozygous *gl15-S*/+ *fdl1-1*/+ F_1_ plants. A first analysis was performed during seedling development through visual scoring of the F_2_ plant phenotypes (Supplementary Fig. S5A). Four phenotypic classes were detected, comprising wild-type, two distinct single-homozygous mutant classes, namely *gl15-S* and *fdl1-1*, respectively, and double-homozygous *gl15-S fdl1-1* plants exhibiting both mutant traits (Supplementary Fig. S5A). The *gl1* phenotype observed was as described in Fig. 2, while *fdl* mutant plants were recognizable for their impaired growth caused by the presence of fusion events between the coleoptile and first leaf (Supplementary Fig. S5A, white arrowheads), and between leaves (La Rocca *et al*., 2015).

**Fig. 5.**
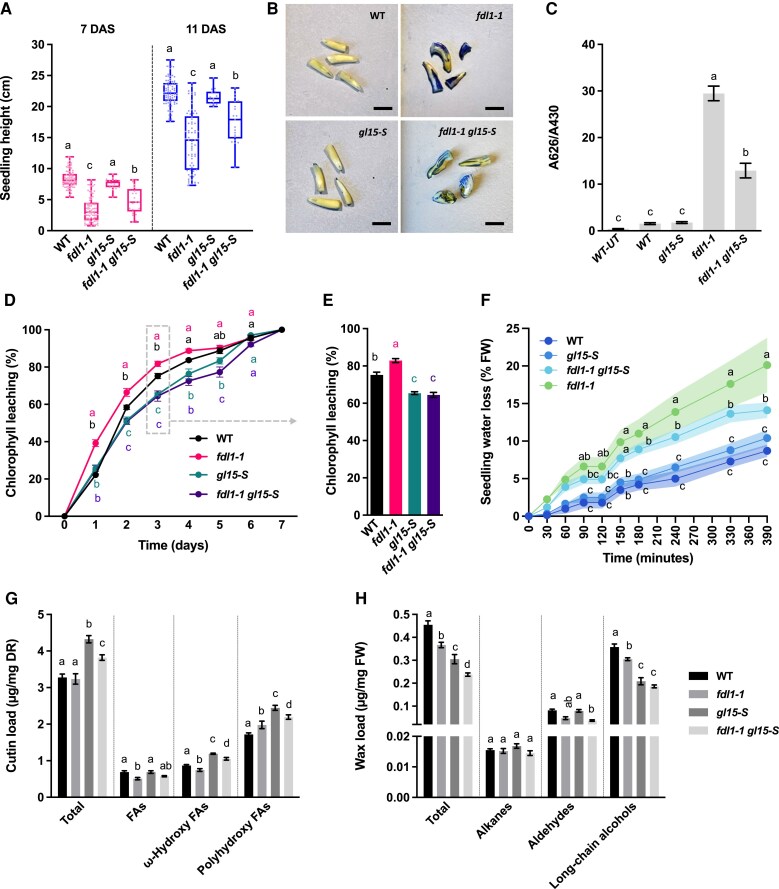
Effects of ZmGL15 and ZmFDL1 on cuticle-related traits. (A) Box plot of seedling height of wild-type (WT), single-homozygous *fdl1-1* and *gl15-S*, and double-homozygous *fdl1-1 gl15-S* plants at 7 (red) and 11 (blue) DAS. Values represent the mean of minimum 15 biological replicates per genotype. (B) Toluidine blue assay in etiolated coleoptiles excised post-staining from 3-old-day (3 DAS) seedling of WT, *gl15-S*, *fdl1-1*, and *fdl1-1 gl15-S*. Bars, 1 cm. (C) Quantification of toluidine blue uptake by the coleoptile of young seedlings, normalized to chlorophyll content. Values represent the average of a minimum of six biological replicates. Error bars represent ±SE. UT, untreated samples. (D) Chlorophyll leaching assay on the second fully expanded leaf of WT, single-homozygous *fdl1-1* and *gl15-S*, and double-homozygous *fdl1-1 gl15-S* plants. Values represent the mean of a minimum four biological replicates per genotype. Error bars are ±SE. The dashed gray rectangle and arrow highlight the time point represented in (E). (E) Cuticle-dependent leaf permeability at day 3 of the assay is shown in (B). Error bars are ±SE. (F) Percentage of water loss in detached 15-day-old homozygous *gl15-S*, *fdl1-1*, *fdl1-1 gl15-S*, and WT seedlings. Values represent the mean of biological replicates. Line shadings represent ±SE. (G) Cutin aliphatic and (H) cuticular wax content of the second fully expanded leaf from 15-day-old plants. Values represent the mean of six biological replicates per genotype ±SE. Different letters denote statistically significant differences between genotypes assessed by Tukey’s HSD test (*P*<0.05).

Concerning seedling height, no differences were detected between the wild type and *gl15-S*, at both 7 and 11 DAS (Fig. 5A). A statistically significant reduction in the elongation was instead observed for both *fdl1-1* and *fdl1-1 gl15-S* as compared with both the wild type and *gl15-S* (Fig. 5A; Supplementary Fig. S5A). This reduction was greater in *fdl1-1* than in *fdl1-1 gl15-S* (Fig. 5A). An in-depth analysis of the *fdl1-1* mutant disclosed a variability in the expressivity of the *fdl* phenotype that was consistent with previous observations (La Rocca *et al*., 2015). We observed variability also in the *fdl1-1 gl15-S* mutants, but with decreased severity of the *fdl* phenotype. Specifically, we detected four mutant categories referred to as mild (i), intermediate (ii), strong (iii), and severe (iv) *fdl* phenotypes, respectively (Supplementary Fig. S5B–D). The latter category (severe; 13.8%) was only detected in the *fdl1-1* mutants (Supplementary Fig. S5C) and not found among the *fdl1-1 gl15-S* mutant plants (Supplementary Fig. S5D). In the double mutant, we also observed a strong increase in the percentage of mild and intermediate *fdl* phenotypes: from 23.9% to 40.2% and from 25.5% to 44.7% for *fdl1-1* and *fdl1-1 gl15-S* mutants, respectively. Consequently, the frequency of the strong phenotype decreased from 36.7% for *fdl1-1*, to 15.1% for *fdl1-1 gl15-S* mutants (Supplementary Fig. S5C, D). Also, comparing single *fdl1-1* and double *fdl1-1 gl15-S* mutant seedlings, both within the category of the intermediate *fdl* phenotype (Supplementary Fig. S5A), we observed that the single *fdl1-1* mutants exhibited more evident fusions between the coleoptile and the first leaf which resulted in a more impaired development of the first leaf (missing/narrow leaf) (Supplementary Fig. S5A).

It is also well known that in the *gl15* mutant, the precocious shift to the adult vegetative phase caused early flowering and the *gl15* mutant plants produced fewer leaves (Moose and Sisco, 1994, 1996; Lauter *et al*., 2005). Accordingly, compared with the wild type, a reduction in the total leaf number in the *gl15-S* mutant was observed at flowering (Supplementary Fig. S5E). The total leaf number was not affected in *fdl1-1*, while the *fdl1-1 gl15-S* mutant exhibited a phenotype similar that of *gl15-S* (Supplementary Fig. S5E). The reduced leaf number suggested a precocious shift from the juvenile to the adult vegetative phase also in the *fdl1-1 gl15-S* mutant.

The analysis of the epidermal traits on the abaxial side of second (Supplementary Fig. S5F–I), third (Supplementary Fig. S5J–M), and fourth (Supplementary Fig. S5N–Q) leaves in all four genotypes corroborated this hypothesis. Toluidine blue staining of epidermal peels revealed that wild-type (Supplementary Fig. S5F, J, N) and *fdl1-1* mutant (Supplementary Fig. S5G, K, O) leaves retained juvenile epidermal traits up to the fourth leaf since all stained pink. In contrast, the epidermis of *gl15-S* and *fdl1-1 gl15-S* mutant plants exhibited pink-stained epidermal cells and juvenile traits in the second leaves only (Supplementary Fig. S5H, I), while showing pink/turquoise staining and turquoise staining in the epidermal tissues of the third (Supplementary Fig. S5L, M) and fourth leaves (Supplementary Fig. S5P, Q), respectively.

The analysis of seedling developmental defects (Fig. 5A; Supplementary Fig. S5A–D) indicated that the *gl15-S* mutant can partially rescue the *fdl* phenotype. Furthermore, analysis of flowering time (Supplementary Fig. S5E) and epidermal traits (Supplementary Fig. S5F–Q) unambiguously demonstrated that *gl15-S* is epistatic to *fdl1-1*.

The accumulation of toluidine blue stain was higher in coleoptiles of single *fdl1-1* mutants compared with *gl15-S fdl1-1* double mutants, whereas it was very low in both *gl15-S* and wild-type coleoptiles (Fig. 5B, C). Further analyses were conducted on the second leaf based on the following observations: (i) differences in cuticle permeability between *fdl1-1* and the wild-type control plants were more pronounced in the second leaf (Castorina *et al*., 2020); (ii) the glossy phenotype appears from the third leaf onwards in the *gl15-S* mutant (Fig. 2A), indicating a maintenance of juvenile epidermal traits in the second leaf (Supplementary Fig. S2E–H); and (iii) despite its dull appearance, the second leaf of the *gl15-S* mutant showed a reduced cuticle-dependent permeability, accompanied by alterations in its chemical cuticle composition and content (Fig. 4).

The chlorophyll leaching assay was conducted on the four genotypes. The results showed that the second leaf of *fdl1-1* mutants released chlorophyll faster than the wild type and *gl15-S* mutants (Fig. 5D, E). Interestingly, chlorophyll leaching was equivalent in the *gl15-S* and *fdl1-1 gl15-S* mutants (Fig. 5D, E). Accordingly, seedling water loss showed similar values in the single *gl15-S* mutant and wild type, but occurred more rapidly in the *fdl1-1* mutant compared with the *fdl1-1 gl15-S* double mutant (Fig. 5F).

### ZmGL15 and ZmFDL1 regulate juvenile cuticle deposition

To elucidate the cuticle alterations that rescued the *fdl1-1* leaf permeability defects in the double *fdl1-1 gl15-S* mutant, the cuticle composition was analyzed on the second fully expanded leaves of wild-type, and *gl15-S*, *fdl1-1*, and *fdl1-1 gl15-S* mutant plants (Fig. 5G, H; Supplementary Fig. S6).

While the wild type and *fdl1-1* mutant had comparable cutin loads, both *gl15-S* and the *fdl1-1 gl15-S* mutants showed a higher total cutin content (Fig. 5G). No statistical differences were detected in the total FA content between genotypes, except for a slight decrease in *fdl1-1* plants. The ω-hydroxy FA content showed a slight reduction in the *fdl1-1* mutant and a significant increase in both *gl15-S* and *fdl1-1 gl15-S* compared with control plants (Fig. 5G). An increase in polyhydroxy FAs was observed in all the mutant genotypes compared with the wild type (Fig. 5G). Overall, these cutin analyses showed that *gl15-S* exhibited the highest contents, and the *fdl1-1 gl15-S* cutin content was higher than in *fdl1-1*, which may be attributed to the epistatic effect exerted by *gl15-S* on *fdl1-1*.

Considering the relative abundance of the different cutin compound classes, differences between genotypes have been detected. All mutant seedling leaves (15–15.9%) exhibited a decrease in the FA percentage compared with the wild type (21%) (Supplementary Fig. S6A). The *fdl1-1* single mutant exhibited the highest (61.1%) and the lowest (23%) relative abundance of polyhydroxy FAs and ω-hydroxy FAs, respectively (Supplementary Fig. S6A). In contrast, the double *fdl1-1 gl15-S* and single *gl15-S* mutants showed statistically comparable levels of polyhydroxy FAs (57.3% and 56.5%) and ω-hydroxy FAs (27.5% and 26.5%), respectively. This is also indicative of an epistatic effect of *gl15-S* on *fdl1-1.* The relative abundances of polyhydroxy FAs in *fdl1-1 gl15-S* and *gl15-S* mutants were also higher than in the wild type (52.4%), while that of ω-hydroxy FAs did not differ from the wild type (26.6%) (Supplementary Fig. S6A).

The epistatic effect of the *gl15-S* recessive allele on *fdl1-1* was also detectable in single cutin metabolites (Supplementary Fig. S6A, C–E). The amount of FA isomers was statistically lower in *fdl1-1* compared with wild-type plants, but in *fdl1-1 gl15-S* the levels were restored to those of the control plants (Supplementary Fig. S6C). Also, considering the ω-hydroxy FA isomers, except for C18:1 and C24:0, the amounts in the double-homozygous *fdl1-1 gl15-S* mutant were equal to those in the wild type even when the amount was lower in the single-homozygous *fdl1-1* mutant (Supplementary Fig. S6D). The amount of the 18:1 ω-hydroxy FAs (18:1ωOH), the most abundant cutin isomer, was the highest in *gl15-*S and was much higher in the double-homozygous *fdl1-1 gl15-*S mutant compared with both wild-type and *fdl1-1* plants (Supplementary Fig. S6D). An increase in the content of several polyhydroxy FA isomers was also observed in the leaves of *gl15-S* and *fdl1-1 gl15-*S mutants (Supplementary Fig. S6E). For the phenolic compounds, we observed significant differences between genotypes: ferulate levels increased in all mutants compared with wild-type plants, while reductions were observed for both coumarate and caffeate (Supplementary Fig. S6F).

Both *gl15-S* and *fdl1-1* mutations caused a significant decrease in total wax load as compared with wild-type plants (Fig. 5H). This decrease was enhanced in the *fdl1-1 gl15-S* mutant, thus suggesting an additive effect of the two genes on wax abundance. Decreases in the *fdl1-1 gl15-S* mutant were also observed when examining specific classes of wax metabolites, except for the total alkane load that did not statistically differ among the four genotypic classes (Fig. 5H). The total aldehyde content was reduced in the double *fdl1-1 gl15-S* mutant compared with the wild type but not in single mutants (Fig. 5H). The abundance of long-chain alcohols, which was diminished by both *gll15-S* and *fdl1-1* mutations, showed equal levels in *fdl1-1 gl15-S* and *gl15-S* mutants (Fig. 5H).

Differences between genotypes were also detected when analyzing the wax relative abundance of a specific class of metabolites. Compared with wild-type plants (3.4%), a progressive increase in alkane percentages was found in *fdl1-1* (4.1%), *gl15-S* (5.5%), and *fdl1-1 gl15-S* (6.1%) (Supplementary Fig. S6B). The relative abundance of aldehydes compared with the wild-type control (17.9%) was statistically lower in the *fdl1-1* mutant (12.6%), statistically higher in the *gl15-S* mutant (26.2%), and partially restored in the *fdl1-1 gl15-S* mutant (15.5%) (Supplementary Fig. S6B). Relative to the total wax abundance, the long-chain alcohols appeared enriched in *fdl1-1* and lower in *gl15-S*, compared with the wild type (78.6%), with a percentage of 83.2% and 68.3%, respectively (Supplementary Fig. S6B). These differences were lost in the *fdl1-1 gl15-S* mutant (78.3%) which was similar to the wild-type control (Supplementary Fig. S6B).

Various trends were observed for the specific wax metabolites (Supplementary Fig. S6G–I). The abundance of C30 and C32 *n*-aldehydes (ALD30 and ALD32; Supplementary Fig. S6G) was not affected by the *gl15-S* mutation and was lower in *fdl1-1* single and *fdl1-1 gl15-S* mutant plants. Additive effects were visible for C24 (C24OH) and C30 (30OH) alcohols which showed the highest concentration in the wild type and progressively lower concentrations in single *fdl1-1* and *gl15-S* and double *fdl1-1 gl15-S* mutant plants (Supplementary Fig. S6H).

**Fig. 6.**
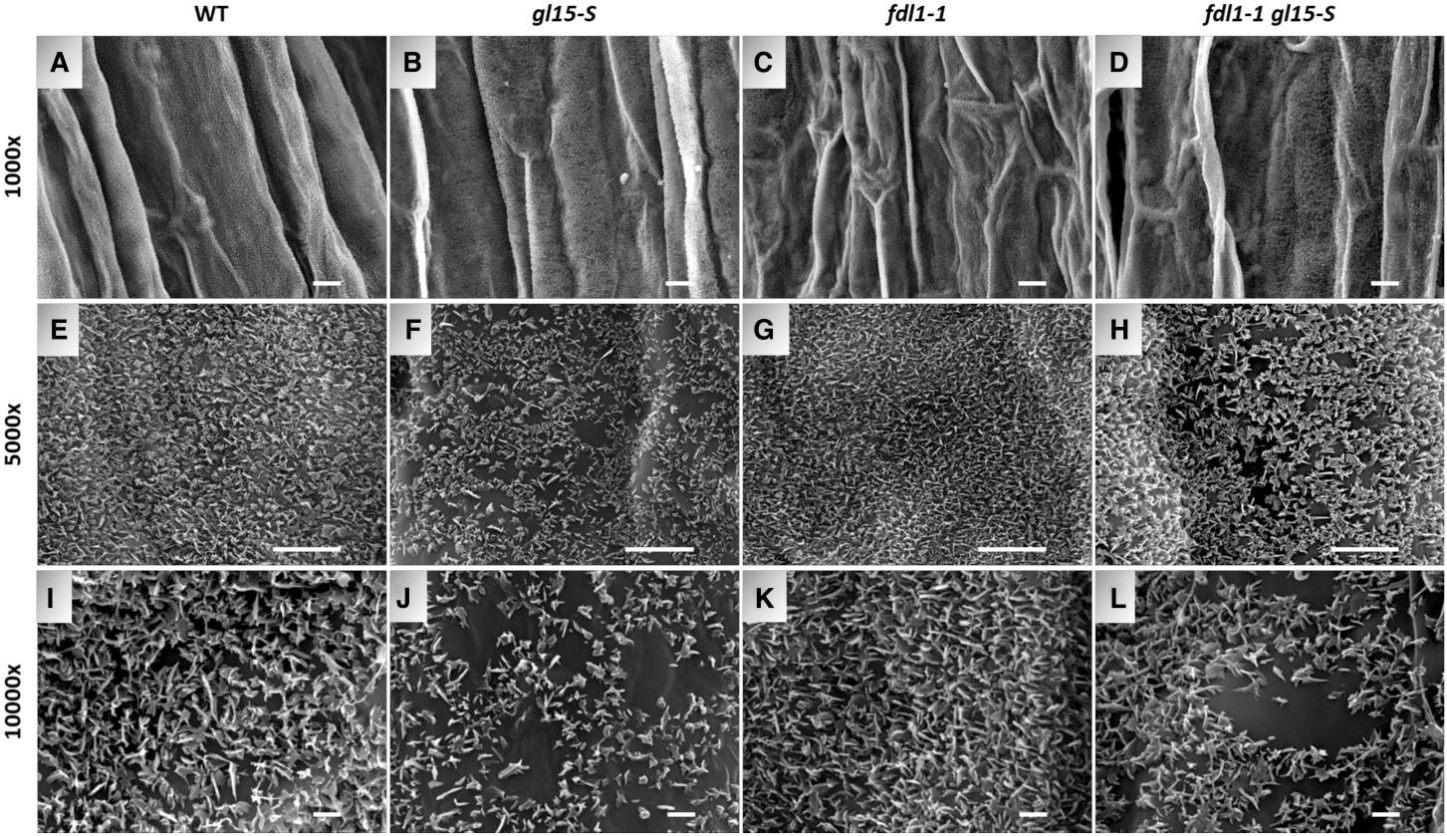
Distribution of cuticular waxes on the adaxial leaf surface of the *fdl1-1 gl15-S* double mutant. SEM micrograph images of the adaxial surface of the second fully expanded leaf in a wild-type (WT) control plant (A, E, I), single-homozygous *gl15-S* (B, F, J), single homozygous *fdl1-1* (C, G, K), and double-homozygous *fdl1-1 gl15-S* (D, H, L) mutants have been acquired at ×1000, ×5000, and ×10 000 magnification. Scale bars correspond to 10 µm (A–D), 5 µm (E–H), and 1 µm (I–L).

To investigate the consequences of the differences in both load and chemical composition on the density and distribution of epicuticular wax crystals, the surface of the second leaf was examined in the four genotypic classes by SEM analysis (Fig. 6; Supplementary Fig. S7). In the *fdl1-1* mutant, no alterations in epicuticular wax crystal density and distribution were detected (Fig. 6C, G, H; Supplementary Fig. S7C, G, H), suggesting that reduction in the total wax content (Fig. 5E) could alter the cuticle-dependent leaf permeability (Fig. 5B) but was not sufficient to induce ultrastructural modifications. In contrast, despite the dull appearance of the second leaf, the *gl15-S* mutant presented fewer wax crystals (Fig. 6J; Supplementary Fig. S7J), as we previously observed in the third glossy leaf (Fig. 3J, L). The epicuticular alterations observed on both adaxial (Fig. 6B, F, J) and abaxial (Supplementary Fig. S7B, F, J) leaf surfaces in *gl15-S* mutants were similar to those of *fdl1-1 gl15-S* mutants (Fig. 6D, H, L; Supplementary Fig. S7D, H, L). Specifically, the surfaces of both *gl15-S* and *fdl1-1 gl15-S* genotypes displayed a lower density of wax crystalloids, and these crystalloids were more irregular and flattened than in the wild-type control (Fig. 6A, E, I; Supplementary Fig. S7A, E, I) and the *fdl1-1* mutant (Fig. 6C, G, H; Supplementary Fig. S7C, G, H). The area of the amorphous regions appeared even more extended in the *fdl1-1 gl15-S* mutant compared with *gl15-S*, supporting the idea of an additive effect of the *ZmFDL1* and *ZmGL15* genes on wax biosynthesis.

Overall, these data showed that in maize both the load and composition of the juvenile cuticle and the ultrastructure of epicuticular wax crystals were influenced by the genetic interaction between the ZmGL15 and ZmFDL1 regulatory factors. The action of the two genes appeared to be exerted in different ways on specific metabolites of both components (wax and cutin) of the cuticle.

## Discussion

### Fine-tuned spatiotemporal expression pattern of *ZmFDL1* and *ZmGL15*

Previous studies have indicated that the *ZmFDL1* gene plays a key role on the control of juvenile cuticle deposition in maize (La Rocca *et al*., 2015; Castorina *et al*., 2020). The single-homozygous *fdl1-1* mutant plants exhibited a defective phenotype in the first stages of growth, whereas, following the third or fourth leaf stage, they resumed a normal phenotype and were indistinguishable from wild-type siblings (La Rocca *et al*., 2015). Accordingly, cuticle-related chemical defects appeared transiently in germinating *fdl1-1* mutant seedlings (up to the second leaf stage) while a progressive shift to control was observed in subsequent plant developmental stages (Castorina *et al*., 2020). We proposed that cuticle deposition during the juvenile phase is due to the interaction between ZmFDL1 and other regulatory factors involved either in maintaining the juvenile phase or in promoting the transition from juvenile to adult phase (Castorina *et al*., 2020). In this context, the *ZmGL15* gene (Moose and Sisco, 1994) was considered as an interesting candidate. In wild-type maize plants, the *ZmGL15* transcript level is progressively down-regulated, as confirmed in Fig. 1A, to promote juvenile-to-adult vegetative phase change (Lauter *et al*., 2005). However, lack of ZmGL15 activity did not completely abolish the juvenile vegetative phase, as the first two leaves of the single homozygous *gl15* mutant plants exhibited characteristic juvenile epidermal traits such as dull leaves (Fig. 2A) and toluidine blue-stained pink epidermal peels (Supplementary Figs S2 and S5).

ZmGL15 was demonstrated to be crucial for the maintenance of the juvenile epidermal traits (Lauter *et al*., 2005). In agreement with this, we show that ZmGL15 controls the expression of cuticle-related genes (Fig. 4C) and in particular that of *ZmFDL1*, which was down-regulated in the *gl15* mutants (Figs 1D, E, 4C). We found that *ZmGL15* is equally transcribed in the different leaf tissues, while in contrast *ZmFDL1* is much more highly expressed in the epidermis, where the cuticle components are synthesized, than in other tissue layers (Liu *et al*., 2021; Fig. 1C). Considering previous findings that *ZmGL15* acts in a cell-autonomous manner to specifically regulate the juvenile leaf epidermal traits (Moose and Sisco, 1994), we hypothesized that in the epidermis the presence of functional ZmFDL1 and ZmGL15 transcription factors is required to stimulate juvenile cuticle deposition. Therefore, the present study further investigated the genetic relationship between these two transcription factors.

ZmGL15 was demonstrated to be crucial for the maintenance of the juvenile epidermal traits (Lauter *et al*., 2005). In agreement with this, we show that ZmGL15 controls the expression of cuticle-related genes (Fig. 4C) and in particular that of *ZmFDL1* which was down-regulated in different tissues of the *gl15* mutants (Figs 1D, E, 4C). We found that *ZmGL15* is equally transcribed in the different leaf tissues (Fig. 1C), while in contrast *ZmFDL1* is much more highly expressed in the epidermis, where the cuticle components are synthesized, than in other tissue layers (Liu et al., 2020; Fig. 1C). Considering previous findings that *ZmGL15* acts in a cell-autonomous manner to specifically regulate the juvenile leaf epidermal traits (Moose and Sisco, 1994), we hypothesized that in the epidermis the presence of functional ZmFDL1 and ZmGL15 transcription factors is required to stimulate juvenile cuticle deposition. Therefore, the present study further investigated the genetic relationship between these two transcription factors.

### A cutin-enriched cuticle restores the wax-dependent cuticular alterations and exhibits increased leaf water-holding capacity

We first obtained evidence that *ZmGL15* plays a crucial role in modulating the properties of the juvenile cuticle. We observed that *gl15-S* first and second leaves exhibited a dull phenotype and juvenile epidermal traits, while the third leaf was in transition and showed a glossy phenotype (Fig. 2A; Supplementary Fig. S2). Therefore, the *gl15-S* mutant appeared as a useful genetic tool to first characterize the differences between juvenile and adult cuticle in maize.

A decrease in the total wax load and several changes in wax composition (Figs 4, 5; Supplementary Figs S4, S6) were observed, confirming the results obtained in a previous study conducted for a wax-related mutant, also named *gl15*, which showed an altered wax composition (Avato *et al*., 1987). This reduction in cuticular waxes was consistent with the fewer and smaller epicuticular wax crystals observed on *gl15-S* surfaces (Figs 3, 6). In addition, lack of *ZmGL15* action led to the down-regulation of different cuticle-related genes, among which *ZmGL3*, encoding a MYB transcription factor that affects the expression of several genes involved in the biosynthesis of VLCFAs (Liu *et al*., 2012), and of *ZmGL13* an ABC transporter gene required for the accumulation of epicuticular waxes (Li *et al*., 2013).

A higher proportion of alkanes (Supplementary Fig. S4B, F), and a reduced abundance of C32 aldehydes (Supplementary Fig. S4G) and long-chain alcohols (Supplementary Fig. S4B, H; Supplementary Fig. S6B) were observed. These changes, along with increases in cutin load and in certain cutin components, such as ω-hydroxy FAs and polyhydroxy FAs (Figs 4A, 5G), made the cutin composition of the *gl15-S* mutant more similar to that of an adult maize leaf (Bianchi *et al*., 1984; Avato *et al*., 1990; Yang *et al*., 1993; Bourgault and Molina, 2025). The increase of ω-hydroxy FAs was correlated with a reduction of cuticle-dependent leaf permeability (Bourgault *et al*., 2020). We also found that *ZmFDL1* had an impact on wax deposition (Fig. 5D, E) and that the two genes had an additive effect on this trait. A partial epistatic effect of *gl15-S* on *fdl1-1* was instead observed for the cutin load (Fig. 5D).

**Fig. 7.**
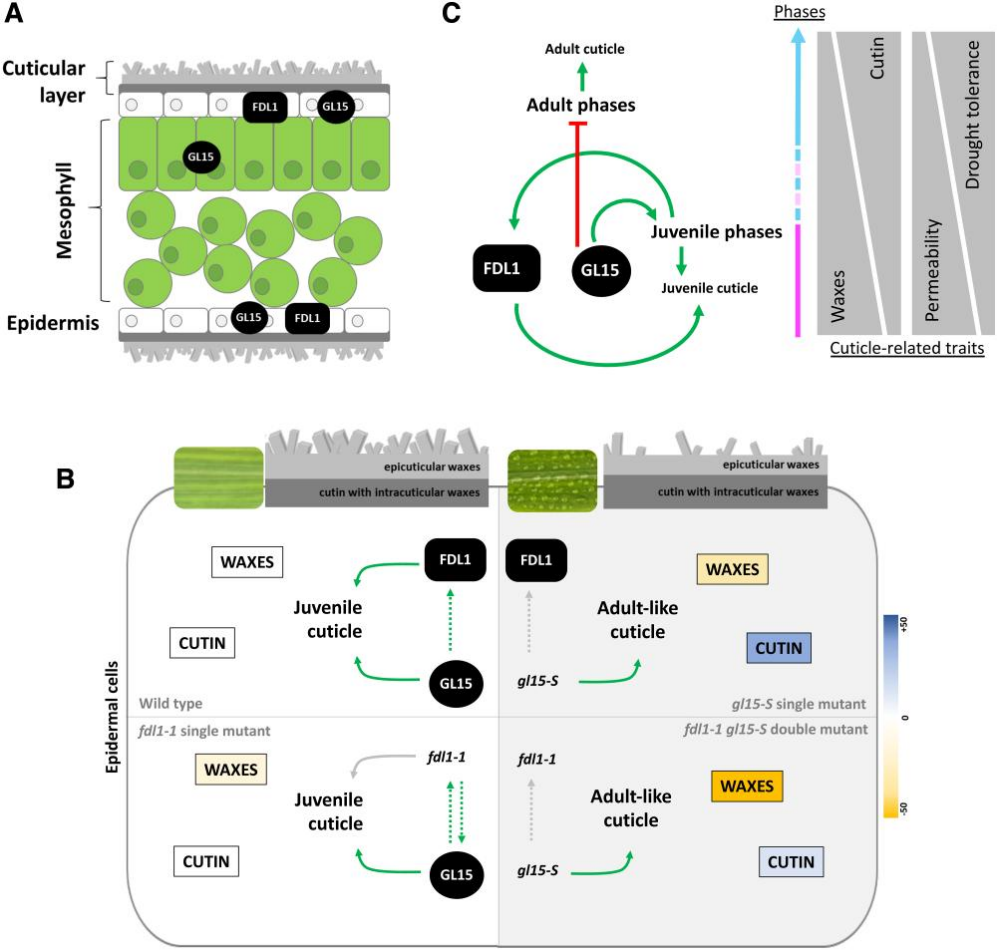
Proposed roles of ZmFDL1 and ZmGL15 transcription factors in the regulation of cuticle deposition in the juvenile leaves. (A) *ZmGL15* is expressed, during the juvenile vegetative phase, in both the epidermal and the mesophyll layer to maintain the juvenile leaf traits. The transcript of *ZmFDL1* is specifically expressed in the epidermis and mainly regulates cuticular wax biosynthesis. (B) Genetic-dependent *ZmFDL1* and *ZmGL15* interaction leads to the proper modulation of juvenile cuticle composition. The cuticle of wild-type plants is rich in waxes which confer the characteristic dull phenotype to the juvenile leaves. Conversely, the adult-like cuticle of the *gl15-S* mutants is poor in waxes, giving the leaves a glossy appearance, but it is also rich in cutin. The color scale reflects the magnitude (±50%) of the total level for each class of compounds. Yellow and blue colors indicate reduced and increased amounts of cuticular compounds with respect to the wild type, respectively. (C) ZmFDL1 and ZmGL15 integrate leaf developmental stimuli in the cuticle biosynthetic pathway, thus ensuring the correct biosynthesis and deposition of the juvenile cuticle. The ZmGL15-mediated establishment of the juvenile phase promotes the expression of *ZmFDL1*. The total wax content decreases in adult leaves compared with juvenile leaves, while the amount of cutin increases. The cuticle-dependent leaf permeability is reduced in adult leaves, thus preventing leaf water loss and improving the tolerance to drought. In (B) and (C), green arrows and red T-shaped lines indicate positive and negative regulation, respectively. Dotted or solid gray arrows represent lost regulation. Dotted lines highlight indirect regulation or compensatory feedback.

Based on these data, a model has been proposed to explain how the genetic interaction between *ZmFDL1* and *ZmGL15* modulates the deposition of the juvenile cuticle. We found that the regulation of *ZmFDL1* and *ZmGL15* transcript levels was dynamic and was dependent on both tissue and phase identity. Their expression varied in different organs (Fig. 1A) and at different developmental stages of the same leaf (Fig. 1B). The expression of *ZmGL15* had been shown to be down-regulated in the *fdl1-1* mutant seedlings at the coleoptile developmental stage (Castorina *et al*., 2020) and, similarly, the *ZmFDL1* transcript level is reduced in the *gl15* mutants (Fig. 1D). This suggests that a reciprocal stimulation occurs between ZmGL15 and ZmFDL1 at early stages of seedling development. At later developmental stages, a different pattern of expression has been observed, suggesting a complex interplay between ZmFDL1 and ZmGL15 (Fig. 1E, F) to regulate cuticle deposition in juvenile leaves. The biosynthesis of a juvenile cuticle, which occurs in the epidermis (Fig. 7A), is positively stimulated by the action of both *ZmFDL1* and *ZmGL15* (Fig. 7B, top left; Fig. 7C), which promotes the biosynthesis of waxes. Lack of the activity of one of the two transcription factors leads to a lower wax load (Fig. 7B, bottom left; top right), and the more pronounced reduction of cuticular wax load in the double *fdl1-1 gl15-S* mutant (Fig. 7B, bottom right) is indicative of an additive effect. Furthermore, we observed an induction of *ZmGL15* expression in the third leaf of the *fdl1-1* mutant (Fig. 1F; Fig. 7B, bottom left), which could represent a feedback compensatory effect to overcome cuticle defects. Our hypothesis is that ZmGL15 action generates a temporal window with high levels of *ZmFDL1* transcripts, thus allowing it to fully express its role in promoting a juvenile cuticle (Fig. 7B, C). ZmGL15 acts as a repressor of cutin deposition, as in its absence cutin is highly synthesized (Fig. 7B, top right). This increase is also observed in the double mutant, but is less conspicuous (Fig. 7B, top left; bottom right), suggesting that ZmFDL1 also positively regulates cutin biosynthesis.

It is important to note that in the absence of *ZmGL15* action, despite the genetic contribution of *ZmFDL1,* the cuticle properties are more similar to those of an adult-like cuticle, with lower wax and higher cutin content (Fig. 7B, top right). The establishment of an adult-like cuticle reduces leaf permeability (Figs 2B, 5B, 5C, 7C; Supplementary Fig. S3A, C) and enhances the leaf RWC under drought stress (Figs 2L, 7C; Supplementary Fig. S3I). This parameter, which reflects the balance between water supply to the leaf tissue and transpiration rate, can be considered as an indicator of the water-holding capacity and water status in plants (Lugojan and Ciulca, 2011). It is therefore conceivable that the less permeable cuticle caused by the *gl15-S* mutation contributes to an increased water-holding capacity of the leaf.

In the absence of *ZmFDL1* action, the juvenile cuticle composition was affected with a decreased total wax load (Fig. 5D, E, H; Fig. 7B, bottom left, Fig. 7C) and was more permeable (Fig. 5B, C). However, the *fdl1-1* defective cuticle-dependent leaf permeability was rescued in the double-homozygous *fdl1-1 gl15-S* mutant (Fig. 5B–E). Our hypothesis is that despite the significant reduction in the total wax load observed in the double mutant, the *gl15*-*S*-dependent high enrichment in cutin is sufficient to overcome the wax alterations and render the cuticle less permeable (Fig. 7B, bottom right, Fig. 7C).

### Cutin is a key component in the constitution of an effective cuticular water barrier in leaf maize seedlings

The cuticle, composed of cutin and cuticular waxes, provides structural integrity to the plant surface. A well-formed cuticle is known to help in maintaining cell turgor and preventing desiccation under drought conditions (Kerstiens, 2006). However, the complex relationships between cuticle load, composition, structure, and water barrier function are still poorly understood.

Most of the studies have considered the role of cuticular wax amount in the response to drought (Cameron *et al*., 2006; Shepherd and Wynne Griffiths, 2006; Bourdenx et al., 2011; Zhou *et al*., 2013; Zhu and Xiong, 2013; Li *et al*., 2019; Lee and Suh, 2022). As regards wax composition, more recently wax esters were shown to be involved in cuticle-mediated leaf permeability in maize (Lin *et al*., 2024).

In maize, few studies have addressed the role of the cutin fraction in leaf permeability. Often, the cuticular waxes are identified as the actual barrier of the plant cuticle against the diffusion of water, and the cutin matrix is considered as a kind of framework within which waxes are deposited and crystallized. It has been proposed that mutations that destroy the cutin framework potentially disrupt wax crystallization, and subsequently the permeability function of the cuticle. However, the dehydration-avoidant ZPBL 1304 maize line exhibited significantly lower rates of leaf water loss and a thicker cuticle compared with the dehydration-susceptible ZPL 389 maize line, but contrary to expectations it had less cuticular waxes (Ristic and Jenks, 2002). These results indicated that water flow through the cuticle is complex and that the amount of cuticular wax alone does not determine the rate of epidermal water loss, suggesting that other factors are involved (Ristic and Jenks, 2002).

The main role of cutin was first confined to plant development where it is responsible for preventing fusion of epidermal surfaces. This was evidenced by the studies of cutin-defective mutants which frequently display organ fusions (Sieber *et al*., 2000; Bird *et al*., 2007; Ingram and Nawrath, 2017). This role was also confirmed by our data: the *gl15*-dependent increase in total cutin load significantly alleviated the developmental defects observed in the *fdl1-1* seedlings (Supplementary Fig. S5C, D). Nevertheless, it is still largely unclear what role cutin plays in cuticle-dependent leaf permeability to restrict transpirational water loss.

Cutin is the most abundant structural component of the cuticle (Pollard *et al*., 2008; Beisson *et al*., 2012). Several transcription factors have been identified (Kannangara *et al*., 2007; Yeats and Rose, 2013; Suh *et al*., 2022) as playing a crucial role in the regulation of the expression of cutin biosynthesis genes (Girard *et al*., 2012; Yeats *et al*., 2012, 2014; Philippe *et al*., 2020).

In tomato, the fruits of the *cutin deficient 1* (*cd1*) mutant showed a significantly increased transpiration rate (Isaacson *et al*., 2009) and the leaf of the *sticky peel* (*pe*) mutant exhibited increased cuticular permeability (Nadakuduti *et al*., 2012). In Arabidopsis, the lower cutin monomer content of the *gpat4*/*gpat8* mutant plants resulted in increased cuticle permeability despite the fact that the load and composition of total cuticular waxes were similar to those of the wild type, suggesting that the increased water loss in the *gpat4/gpat8* plants cannot be attributed to waxes *per se* (Li *et al*., 2007). This was also consistent with previous observations on the *att1* and *lacs2* cutin mutants (Schnurr *et al*., 2004; Xiao *et al*., 2004) and thus confirmed that cutin is important for water barrier function. More recent evidence has highlighted that, under drought stress, the expression of genes involved in cutin biosynthesis is often up-regulated, and a functional cutin is essential to cope with environmental stresses (Chen *et al*., 2011; Fich *et al*., 2016). The importance of cutin for the water barrier function of the cuticle is also substantiated by the comparison of the cuticle composition in maize adult leaves at various developmental stages, which showed that cutin amount correlated with cuticle-dependent leaf transpiration (Bourgault *et al*., 2020). The reduction in cuticle permeability during leaf development coincides with an increase in the deposition of the cutin polyester, thus implying that the cutin-rich layer is a key component to establish the water barrier property of the mature cuticle (Bourgault *et al*., 2020).

Moreover, cuticle amount and composition are modulated in response to environmental changes. In Arabidopsis, water deficit-treated plants showed 75% more wax than control plants and a 65% increase in total cutin (Kosma *et al*., 2009). The predominant alkanes accounted for the observed large increase in total wax amount, whereas nearly all cutin monomers were increased. This indicates that both the cuticular wax composition and load, and the total cutin load, rather than the amount of any specific cutin constituent, are of importance in the water deficit stress response (Kosma *et al*., 2009).

A more recent study in Arabidopsis investigated the role of cuticular waxes, but not cutin, under environmental stresses, proposing a model where the allocation of wax precursors between alcohol and alkane pathways allows the plants to adapt their cuticular wax composition in response to drought (Li *et al*., 2025). The *soh1-1* mutant is characterized by a decrease in primary alcohol levels and an increase in alkane levels, which lead to drought tolerance, thus demonstrating that the amount of primary alcohols negatively correlated with cuticular transpiration (higher water loss) whereas the amount of alkanes positively correlated with drought tolerance (reduced cuticle permeability) (Li *et al*., 2025).

In our study, we showed that a cutin-enriched cuticular layer (Fig. 4A) combined with changes in specific wax types, such as alkanes and primary alcohols (Fig. 4B), in *gl15-S* plants improves the properties of the cuticle (Fig. 2E), thus forming a more effective hydrophobic barrier that limits water loss, thereby helping the plant in retaining moisture (Fig. 2L). Our results also suggest that a cuticular layer with a greater cutin load is more efficient, at least in maize seedlings, in improving the cuticle-dependent permeability and in preventing leaf water loss (Fig. 5B–F). Because waxes are embedded in cutin, the structural organization of the polyester may also be influenced. Therefore, their precise functional assembly is the key point in formation of an effective barrier (Bourgault *et al*., 2020) and in determining non-stomatal leaf transpiration. However, structural organization and assembly of cutin and cuticular waxes, both intra- and epi-cuticular, is very complex to address.

Moreover, our data indicate that an improved cuticle, with an increased amount of cutin, is also able to compensate the leaf permeability alterations due to severe wax reduction. On this basis, we propose that cutin represents a crucial component of the cuticle in establishing an effective water barrier layer and consequently has a positive effect on plant adaptation to water scarcity conditions. These findings provide insights into the complex relationships between cuticle amount, composition, structure, and function in maize leaves, highlighting the importance of specific lipid components in establishing the water barrier property. They corroborate a first idea, which refuted that cuticle permeability depends only on wax load or cuticle thickness (Riederer and Schreiber, 2001).

There is an increasing interest in breeding for cuticle-related traits that promote drought tolerance. Improving cuticle composition can reduce water loss and better protect plants from desiccation, which is crucial for maintaining crop productivity in the context of climate change. Drought tolerance in crops such as wheat, maize, and rice, which are staple foods for a large portion of the global population, can also lead to more stable yields in regions prone to water scarcity (Busta *et al*., 2021; Kan *et al*., 2022). The insights gained from this research can drive the selection of desirable traits in crop breeding, speeding up the development of stress-resistant varieties.

## Supplementary Material

eraf265_Supplementary_Data

## Data Availability

The primary data supporting this study were not made publicly available at the time of publication. Data will be made available upon request to the corresponding author.

## Supplementary data

The following supplementary data are available at *JXB*  online.

Table S1. Primers used for quantitative real-time PCR analysis.

Fig. S1. Expression pattern of *ZmFDL1* and other cuticle genes.

Fig. S2. Epidermal traits in *gl15-S* and wild-type leaves.

Fig. S3. Cuticle-dependent leaf permeability on homozygous *gl15-S* and wild-type control plants.

Fig. S4. Detailed cuticle composition in first, second, and third leaves of the *gl15-S* mutant.

Fig. S5. Epistatic interaction between *ZmGL15* and *ZmFDL1*.

Fig. S6. Detailed cuticle composition in the second fully expanded leaf from 15-day-old plants.

Fig. S7. Distribution of cuticular waxes on the abaxial leaf surface of the *fdl1-1 gl15-S* double mutant.
