# Supplementary material for: Genetic interaction between GL15 and FDL1 modulates juvenile cuticle deposition and leaf permeability in maize
Source: J Exp Bot. 2025 Jun 18;76(18):5285–304. doi: 10.1093/jxb/eraf265 (PMC12596131; doi:10.1093/jxb/eraf265)
Supplement: eraf265_Supplementary_Data [file eraf265_supplementary_data.pdf]

Pathway	Gene model	Gene product	Gene Name	Gene Symbol	Forward Sequence (5'- 3')	Reverse Sequence (5'- 3')	Use	Oligo References
	Zm00001eb385900	Elongation factor 1-alpha subunit	*Elongation factor1α9*	*EF1α**	TGGGCCTACTGGTCTTACTACTG	ACATACCCACGCTTCAGATCCT	RT-qPCR	Lin et al. 2014
Cutin biosynthesis	Zm00001eb042160	fatty acid omega-hydroxy dehydrogenase	*Hothead*	*HTH1*	TACCCAAGCACACAGACGAC	CCCCCATGTATCTGCCCATC	RT-qPCR	Castorina et al 2020
	Zm00001eb311010	fatty acid omega-hydroxy dehydrogenase	*Onion*	*ONI3*	CTGCTGATGGTGCTGGGTTA	GGTTGGTCCCTGGCGATTTA	RT-qPCR	Castorina et al 2020
DNA-binding activity	Zm00001eb328280	R2R3-MYB trascription factor	*Fused leaf1*	*FDL1*	TAGCTGTTCAGATCGGTCG	CCACACAACATGCAACTTGC	RT-qPCR	La Rocca et al. 2016
					GGTAAAAGCAGTGGCAGAGG	TAACTAGTGGCCACCCCAAG	RT-qPCR	Castorina et al 2020
	Zm00001eb195850	R2R3-MYB trascription factor	*Glossy3*	*GL3*	TCTCCTCCAAGGACACCTACTAC	CGGTGAGTAGCCTGGATATGTT	RT-qPCR	
Fatty acid elongation	Zm00001eb246270	beta-ketoacyl reductase	*Glossy8a*	*GL8*	AGCTACCATTACCTCCTGAGTCT	GCAGGCAAAGAGACTTCACAAA	RT-qPCR	
VLCFA biosynthesis	Zm00001eb190120	3-ketoacyl-CoA synthase	*Glossy4a*	*GL4*	GGTACGAGCTCGCCTACATC	CAGGCTTGTAGTCGGGTCAG	RT-qPCR	
	Zm00001eb018600	3-ketoacyl-CoA synthase	*Ketoacyl-CoA synthase39*	*KCS39*	TTCAGCTCCTCTGCTTACGG	TAGTCCAGGAGGTAGCACCG	RT-qPCR	
	Zm00001eb296230	3-ketoacyl-CoA synthase	*Ketoacyl-CoA synthase16*	*KCS16*	CCATGATCGTGAACAAGTACAAGC	GTTGGGGATCAGCATCGACT	RT-qPCR	
Wax biosynthesis	Zm00001eb176110	alcohol-forming fatty acyl-CoA reductases	*Male sterile protein homolog1*	*CER4*	CGGCTCTACAACGACCTCAA	CCTTAGCCGCTCCAGGTTTA	RT-qPCR	Castorina et al 2020
	Zm00001eb247450	fatty acid hydroxylase	*Eceriferum1*	*CER1*	TGTGGTGGTACATGTGGGTG	CAGGCCGTACTGGAAGTTGT	RT-qPCR	Castorina et al 2020
	Zm00001eb156680	O-acyltransferase	*Wax Synthase/Diacylglycerol Acyltransferase*	*WSD1*	TCTAGCGCTCTGTCTCGGTA	AGCATGTAGCCCAGCTTGTT	RT-qPCR	Castorina et al 2020
	Zm00001eb313510	Fatty acid hydroxylase	*Glossy1*	*GL1*	AAGGACGGCATCAACAGACA	CCTTGGGGATCTCGTTCAGG	RT-qPCR	
	Zm00001eb071110	BAHD acyltransferase	*Glossy2*	*GL2*	GTAGCGTAATGAGGCGTGACT	CGATTGTTTTTGTCTAGTCTCCAGT	RT-qPCR	
	Zm00001eb122470	ABC transporter	*Glossy13*	*GL13*	ACCATTGCGCCTATTATTGC	CCGAACTGAGAGGTCAGGAG	RT-qPCR	Li et al. 2013
	Zm00001eb087050	casparian-strip-membrane-domain-like	*Glossy14*	*GL14*	AGCAGAAGACAATACAAAGATGTCC	TGCTGGTTACGGAGCATGAC	RT-qPCR	

Supplemental Table S1. Primers used for quantitative Real-Time PCR analysis. The gene models are referred to the Zm-B73-REFERENCE-NAM-5.0 reference genome version. The asterisk on the EF1a symbol indicates the gene used as housekeeping to normalize the gene expression in RT-qPCR assay.

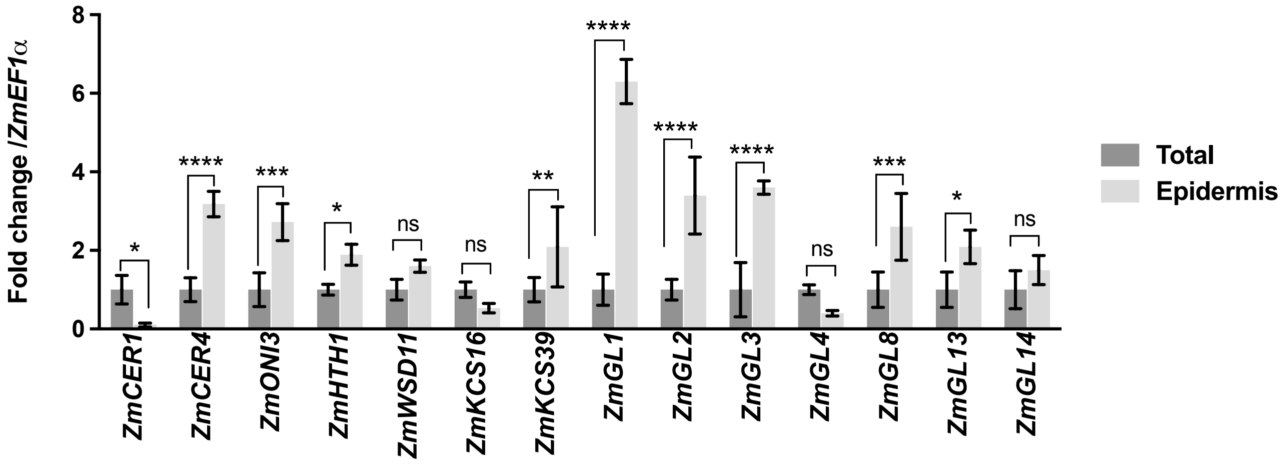

Supplementary Figure 1. Expression pattern of *ZmFDL1* and other cuticle genes. Epidermis enrichment test of cuticle-related gene expression. Transcript level analysis of *ZmCER1, ZmCER4, ZmONI3, ZmHTH1, ZmWSD11, ZmKCS16, ZmKCS39, ZmGL1, ZmGL2, ZmGL3, ZmGL4, ZmGL8, ZmGL13* and *ZmGL14* genes was performed, by RT-qPCR, on the whole leaf (Total) and on the manually dissected (by peeling) epidermal tissue from the second leaves of 10-day-old wild-type plants. Values represent the mean fold change of a minimum of four biological replicates. Error bars are ±SE. Comparison was made between genotypes and significant differences were assessed by Student's T-test (* P<0.05; ** P<0.01; *** P<0.001; **** P<0.0001; ns=not significant).

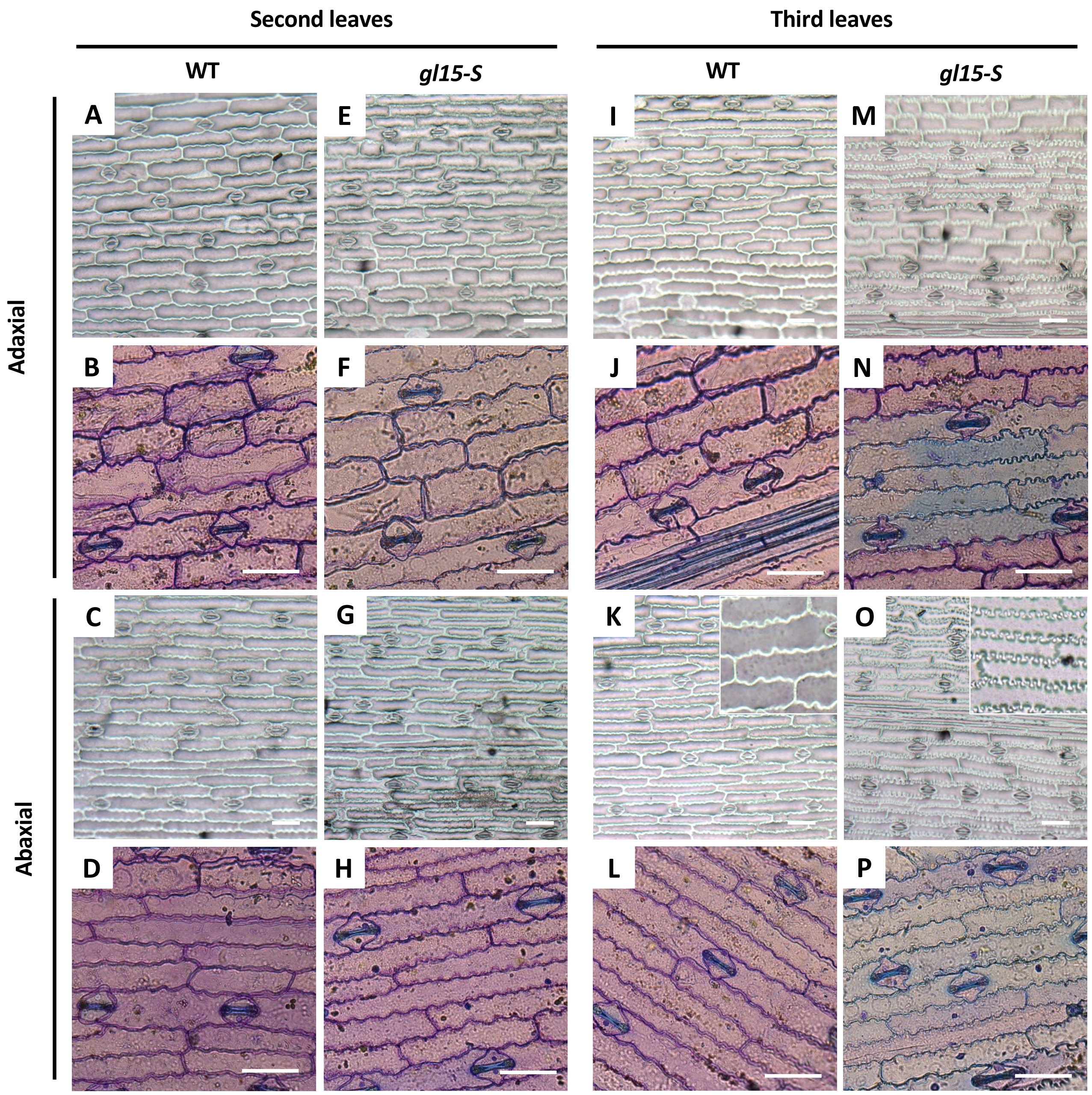

Supplementary Figure 2. Epidermal traits in *gl15-S* and wild-type leaves. Glue-imprinted leaf surface (A, C, E, G, I, K, M, O) and Toluidine blue-stained epidermal peels (B, D F, H, J, L, N, P) from the adaxial and abaxial side of the second and third fully expanded leaves in wild-type (WT) (A-D, I-L) and homozygous *gl15-S* mutant (E-H, M-P) plants. Juvenile epidermal cells uniformly stain violet/pink, and possess wavy cell walls (K, inset). Epidermal cells with adult traits show aqua/turquoise-staining cells with highly crenulated lateral walls (O, inset). Transition leaves present epidermal cells with both juvenile and adult characteristics. Images were acquired with light microscopy. Scale bars correspond to 100 µm (A, C, E, G, I, K, M, O) and 50 µm (B, D F, H, J, L, N, P).

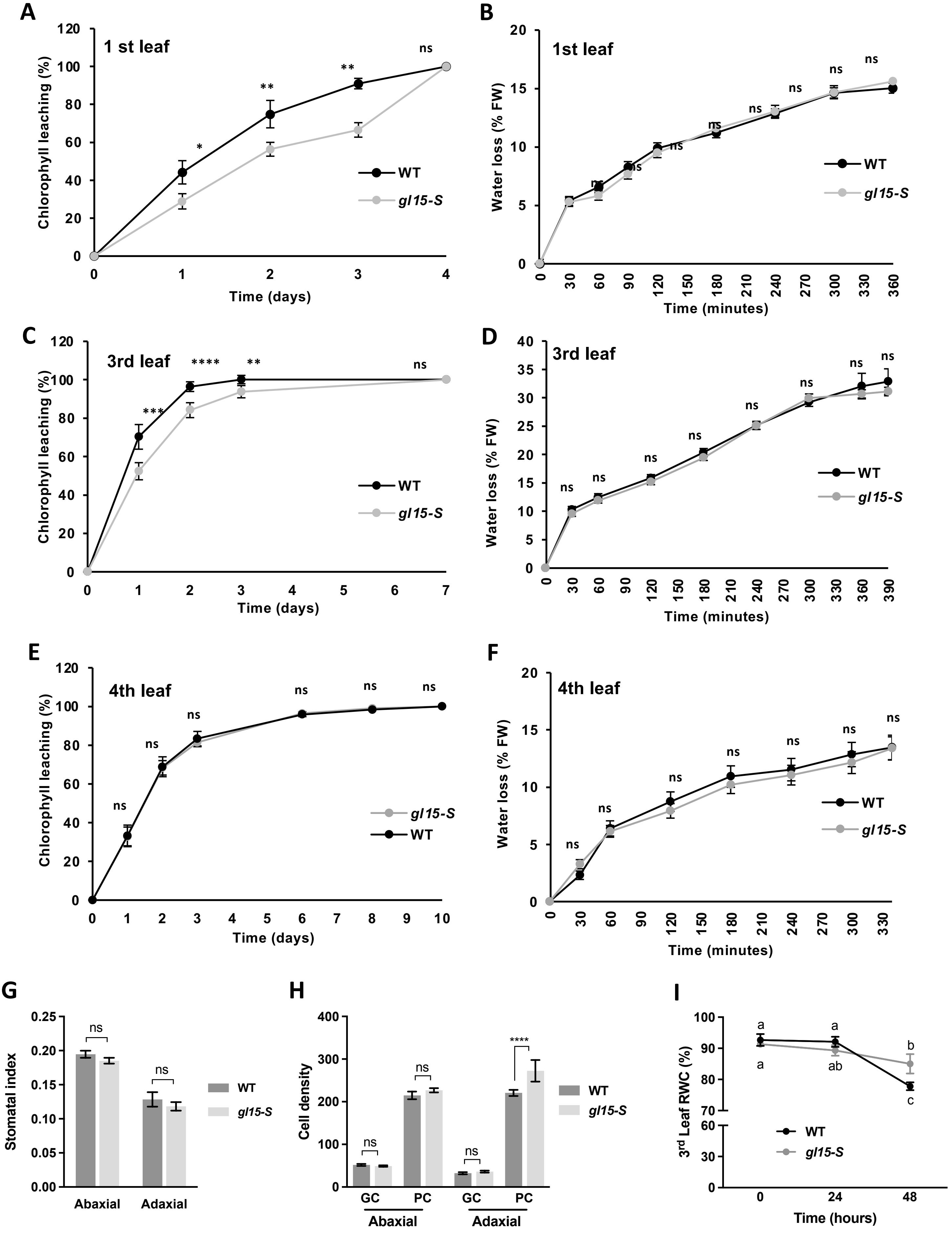
A
1 st leaf
Chlorophyll leaching (%)
Time (days)
WT
gl15-S
*
**
**
ns
B
1st leaf
Water loss (% FW)
Time (minutes)
WT
gl15-S
ns
C
3rd leaf
Chlorophyll leaching (%)
Time (days)
WT
gl15-S
***
****
**
ns
D
3rd leaf
Water loss (% FW)
Time (minutes)
WT
gl15-S
ns
E
4th leaf
Chlorophyll leaching (%)
Time (days)
gl15-S
WT
ns
F
4th leaf
Water loss (% FW)
Time (minutes)
WT
gl15-S
ns
G
Stomatal index
Abaxial
Adaxial
WT
gl15-S
ns
H
Cell density
GC
PC
Abaxial
Adaxial
WT
gl15-S
ns
****
I
3rd Leaf RWC (%)
Time (hours)
WT
gl15-S
a
ab
b
c

Supplementary Figure 3. Cuticle-dependent leaf permeability on homozygous *gl15-S* and wild-type control plants. The chlorophyll leaching assay was performed on the first (A), third (C) and fourth (E) fully expanded leaves of homozygous *gl15-S* and wild-type (WT) control plants. Leaf evapotranspiration in the first (B), third (D) and fourth (F) detached leaves is expressed as the percentage of water loss of fresh weight (FW). Values represent the mean ± SE of 10 biological replicates per genotype. Significant differences were assessed by Student's T-test (*P<0.05; **P< 0.01; ***P<0.001; and ****P<0.0001; ns, not significant). (G) Stomatal index and (H) the density per 1 mm^2 of guard cells (GC) and pavement cells (PC) were measured in both the abaxial and adaxial sides of the third leaf of wild-type (WT) and *gl15-S* plants. Values are the mean ± SE and differences were evaluated by Student's T-test. (I) The relative water content (RWC) was measured in the third leaf of wild-type (WT) and *gl15-S* plants subjected to 24 and 48 hours of water scarcity imposed by withholding irrigation. Values represent the mean of a minimum of four biological replicates. Error bars are ±SE. Different letters denote significant differences assessed by Tukey's HSD test (P <0.05).

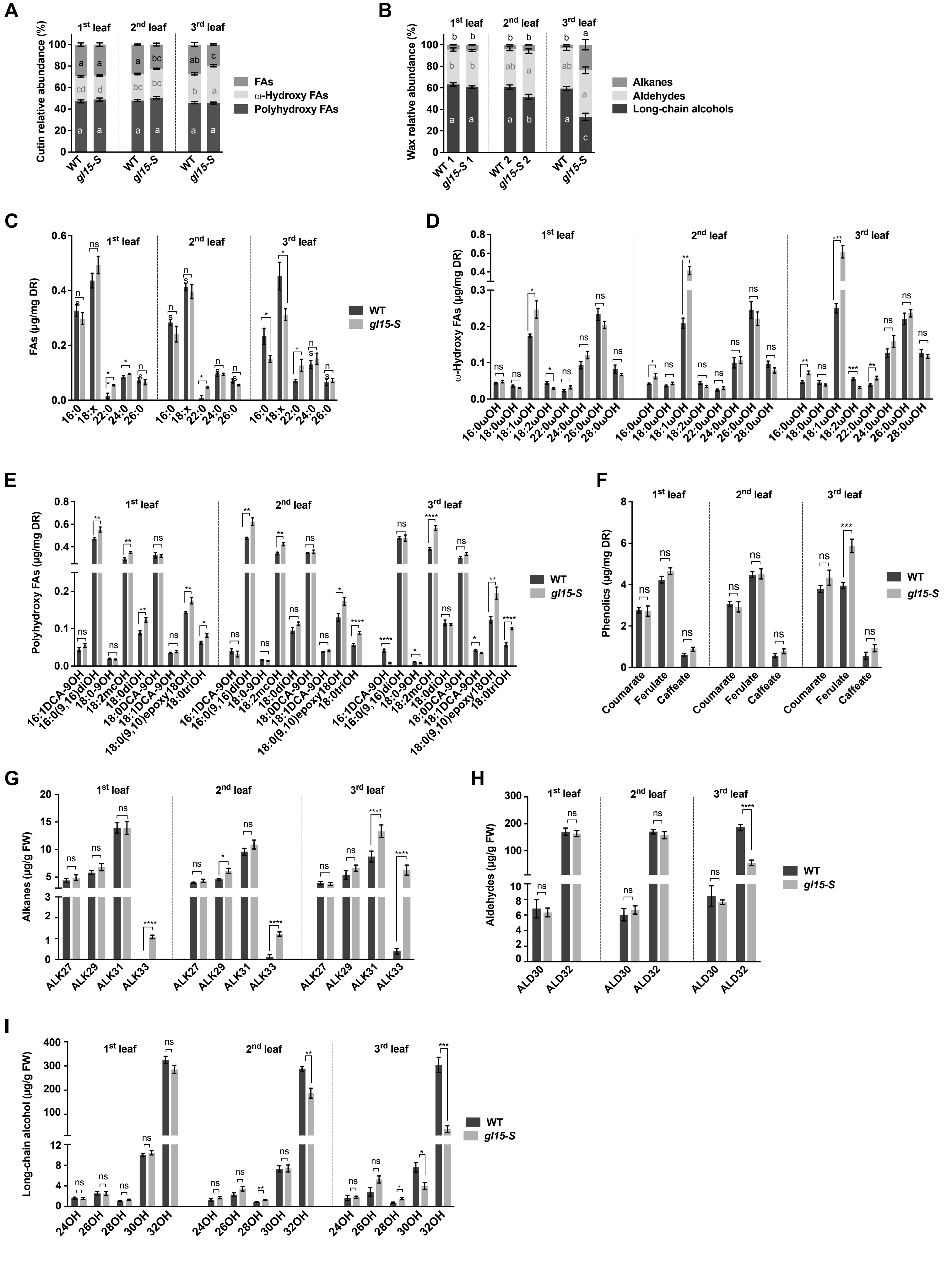

A
1st leaf
2nd leaf
3rd leaf
Cutin relative abundance (%)
FAs
ω-Hydroxy FAs
Polyhydroxy FAs
WT
gl15-S
B
Wax relative abundance (%)
Alkanes
Aldehydes
Long-chain alcohols
WT 1
gl15-S 1
WT 2
gl15-S 2
C
FAs (µg/mg DR)
16:0
18:x
22:0
24:0
26:0
D
ω-Hydroxy FAs (µg/mg DR)
16:0ωOH
18:0ωOH
18:1ωOH
18:2ωOH
22:0ωOH
24:0ωOH
26:0ωOH
28:0ωOH
E
Polyhydroxy FAs (µg/mg DR)
16:1DCA-9OH
16:0(9,16)diOH
18:0-9OH
18:2mcOH
18:0diOH
18:0DCA-9OH
18:1DCA-9OH
18:0(9,10)epoxy18OH
18:0triOH
F
Phenolics (µg/mg DR)
Coumarate
Ferulate
Caffeate
G
Alkanes (µg/g FW)
ALK27
ALK29
ALK31
ALK33
H
Aldehydes (µg/g FW)
ALD30
ALD32
I
Long-chain alcohol (µg/g FW)
24OH
26OH
28OH
30OH
32OH

Supplementary Figure 4. Detailed cuticle composition in first, second and third leaves of *gl15-S* mutant. Percentage of (A) cutin and (B) wax compound classes relative to the total abundance. Black, white and grey letters above or within the data bars indicate statistically significant differences between genotypes as assessed by Tukey's HSD test ($P<0.05$) for comparisons within a specific class of cuticular compound. Relative amounts of (C) fatty acids (FAs), (D) ω-hydroxy fatty acids, (E) polyhydroxy-fatty acids, (F) phenolics, (G) alkanes, (H) aldehydes and (I) long-chain primary alcohols. Significant differences were assessed by Student's T-test (* $P<0.05$, ** $P<0.01$, *** $P<0.001$, **** $P<0.0001$, ns=not significant). Values represent the mean of independent biological replicates (N=6) for wild-type (WT) and single homozygous *gl15-S* mutant plants. Error bars are ±SE.

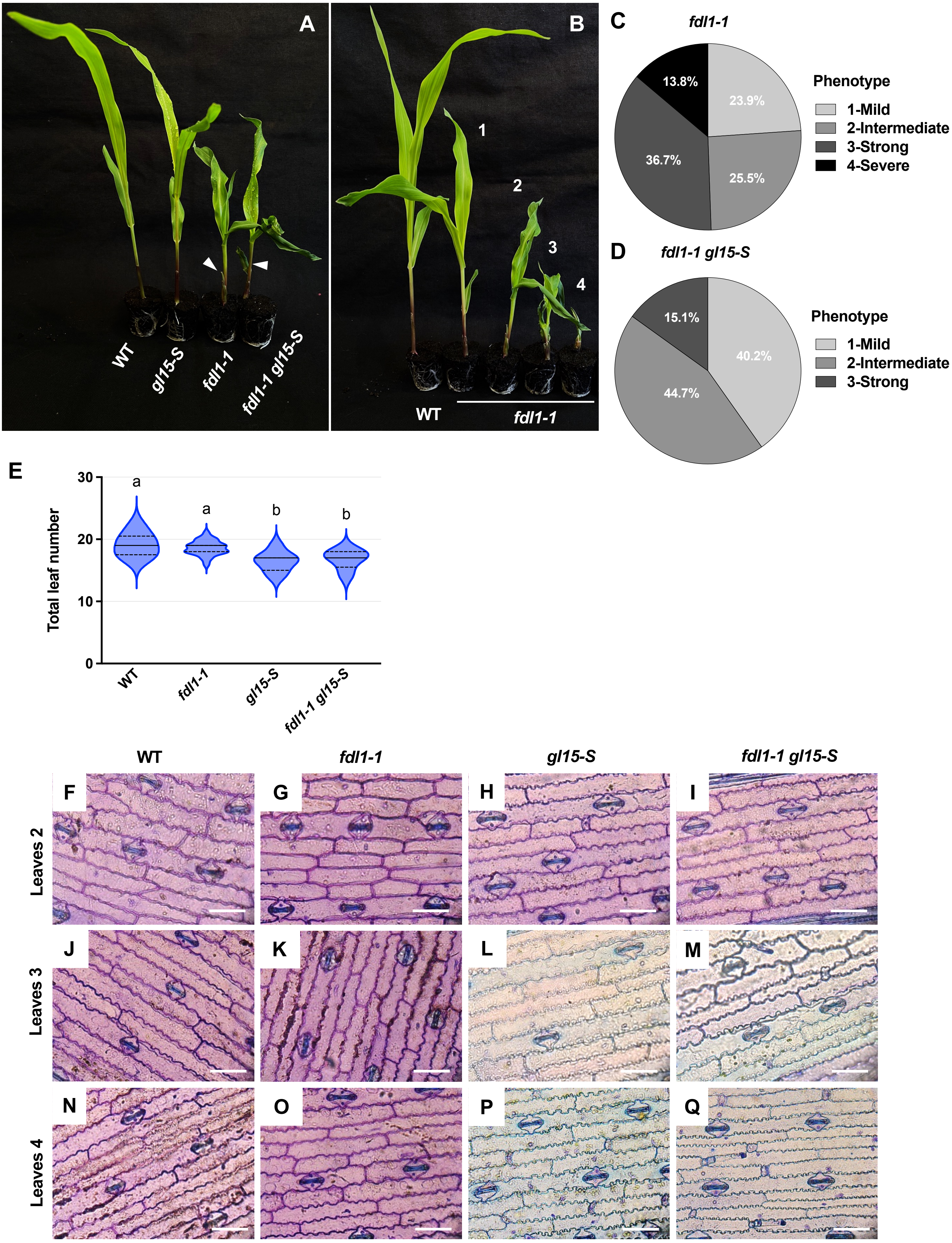

A
B
WT
gl15-S
fdl1-1
fdl1-1 gl15-S
1
2
3
4
WT
fdl1-1
C
fdl1-1
13.8%
23.9%
36.7%
25.5%
Phenotype
1-Mild
2-Intermediate
3-Strong
4-Severe
D
fdl1-1 gl15-S
15.1%
40.2%
44.7%
Phenotype
1-Mild
2-Intermediate
3-Strong
E
Total leaf number
30
20
10
0
a
a
b
b
WT
fdl1-1
gl15-S
fdl1-1 gl15-S
WT
fdl1-1
gl15-S
fdl1-1 gl15-S
Leaves 2
Leaves 3
Leaves 4
F
G
H
I
J
K
L
M
N
O
P
Q

Supplementary Figure 5. Epistatic interaction between *ZmGL15* and *ZmFDL1*. (A) Representative phenotypes of 15-day-old wild-type (WT) control plant, *gl15-S*, *fdl1-1* and *fdl1-1 gl15-S* mutant seedlings. Withe arrowheads indicate fusion in coleoptile and first leaf. (B) Representative phenotype of 15-day-old wild-type (WT) and *fdl1-1* seedlings. Numbers 1, 2, 3 and 4 represent mild, intermediate, strong and severe fdl1 phenotypes, respectively. (C-D) The pie charts reported the frequency of each phenotypic class present in (C) single homozygous *fdl1-1* and (D) double homozygous *fdl1-1 gl15-S* mutant. (E) Box plot of the flowering time expressed as total leaf number measured at maturity. Values represent the mean of independent biological replicates per genotype (minimum N=9). Different letters denote significant differences between genotypes assessed by Tukey's HSD test (P <0.05). (F-Q) Toluidine blue-stained epidermal peels dissected from the abaxial side of the second (F-I), third (J-M) and fourth (N-Q) fully expanded leaves in wild-type (F, J, N), single homozygous *fdl1-1* (G, K, O) and *gl15-S* mutant (H, L, P), and double homozygous *fdl1-1 gl15-S* mutant (I, M, Q) plants. Scale bars correspond to 50 µm.

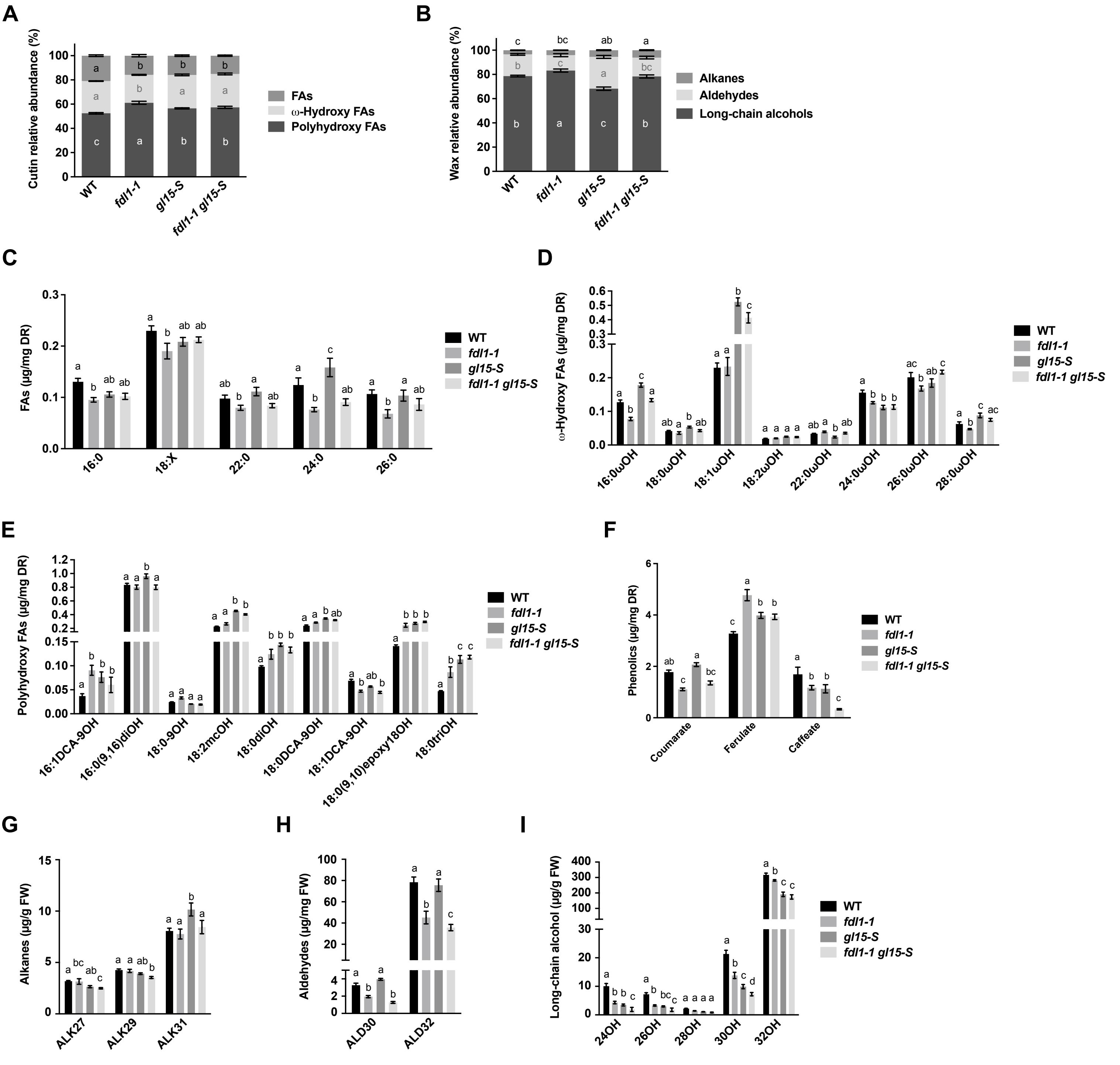

Supplementary Figure 6. Detailed cuticle composition in the second fully expanded leaf from 15-day-old plants. Percentage of (A) cutin and (B) wax compound classes relative to the total abundance. Relative amounts of (C) very-long-chain fatty acids (VLCFAs), (D) ω-hydroxy fatty acids, (E) polyhydroxy-fatty acids, (F) phenolics, (G) alkanes, (H) aldehydes and (I) long-chain primary alcohols. Values represent the mean of independent biological replicates (N=6) for wild-type (WT), single homozygous *fdl1-1* and *gl15-S,* and double homozygous *fdl1-1 gl15-S* mutant plants. Error bars are ±SE. Different letters above data bars denote statistically significant differences between genotypes assessed by Tukey's HSD test ($P < 0.05$). In panels A and B, black, white and grey letters above or within the data bars represent comparisons within a given class of cuticular compound.

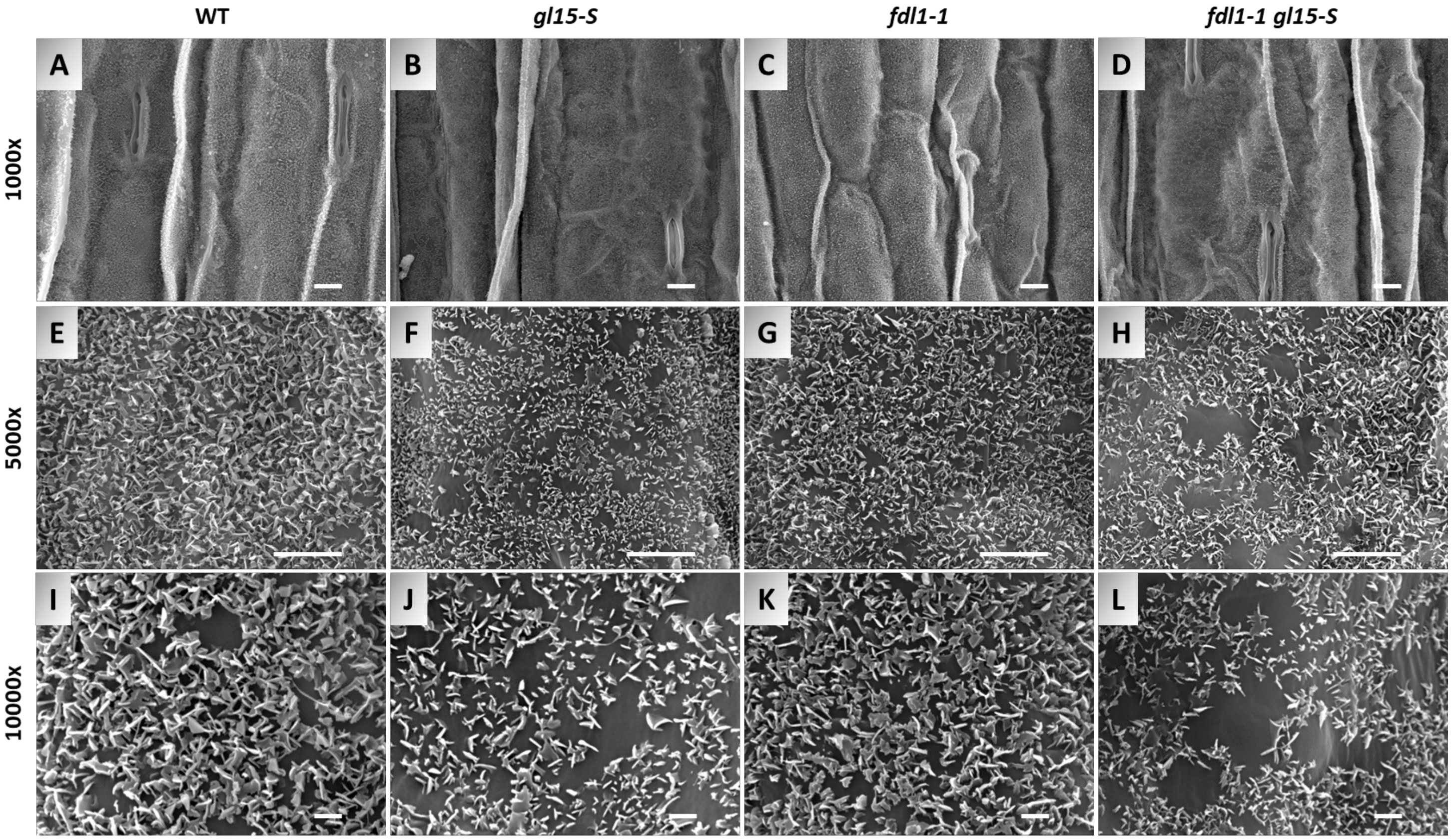

Supplementary Figure 7. Distribution of cuticular waxes on the abaxial leaf surface of the *fdl1-1 gl15-S* double mutant. SEM micrograph images of the abaxial surface of the second fully expanded leaf in wild type (WT) control plant (A, E, I), single homozygous *gl15-S* (B, F, J), single homozygous *fdl1-1* (C, G, K) and double homozygous *fdl1-1 gl15-S* (D, H, L) mutants have been acquired at 1000x, 5000x and 10000x magnification. Scale bars correspond to 10 µm (A-D), 5 µm (E-H) and 1 µm (I-L).
